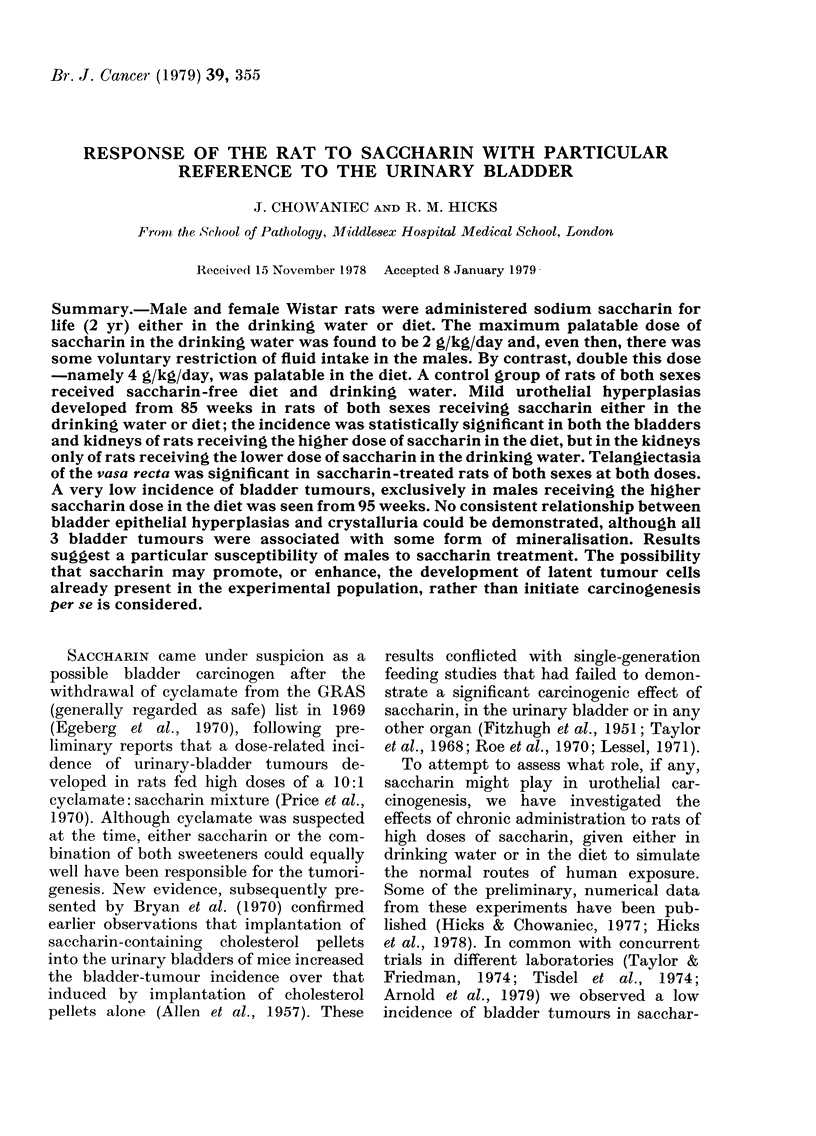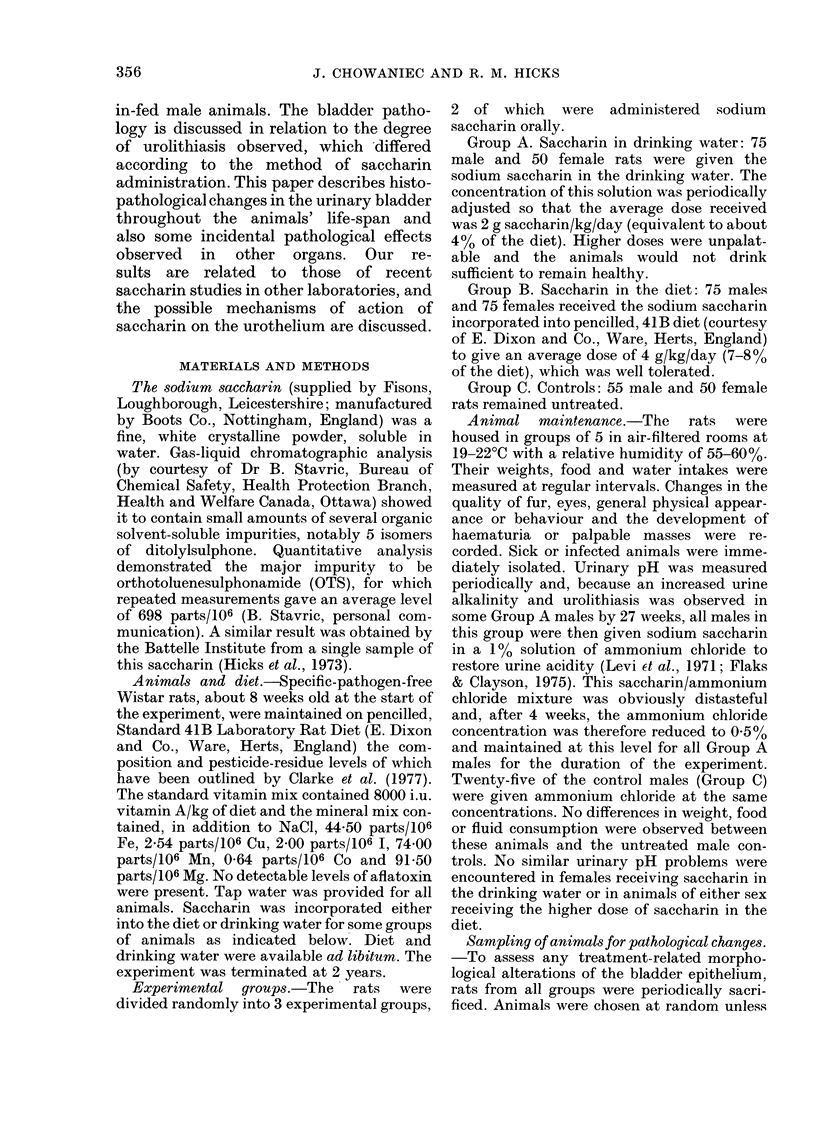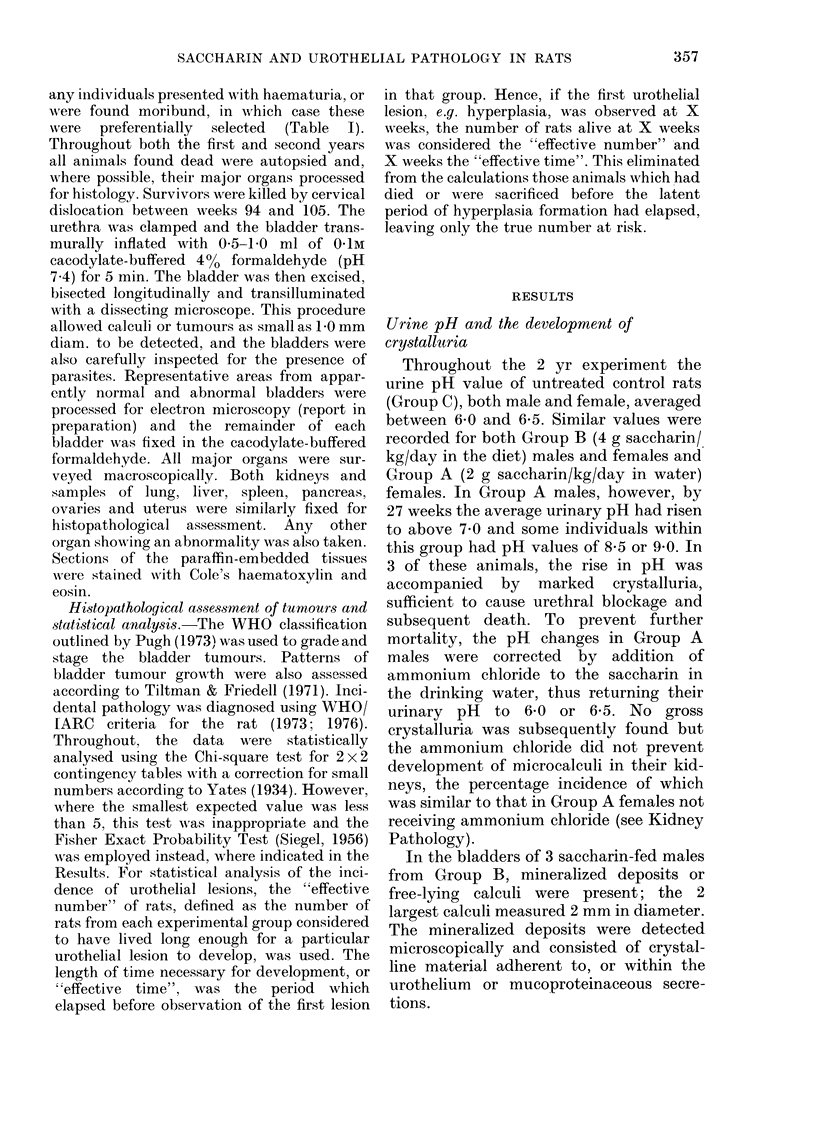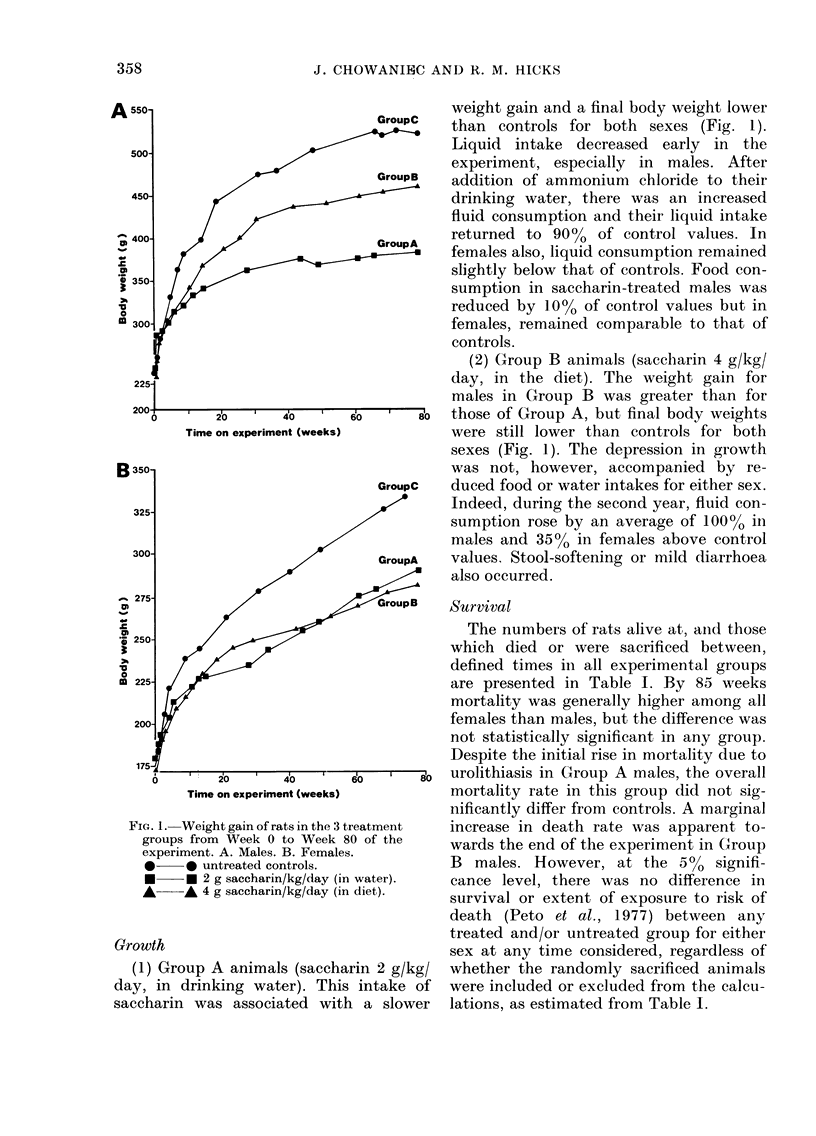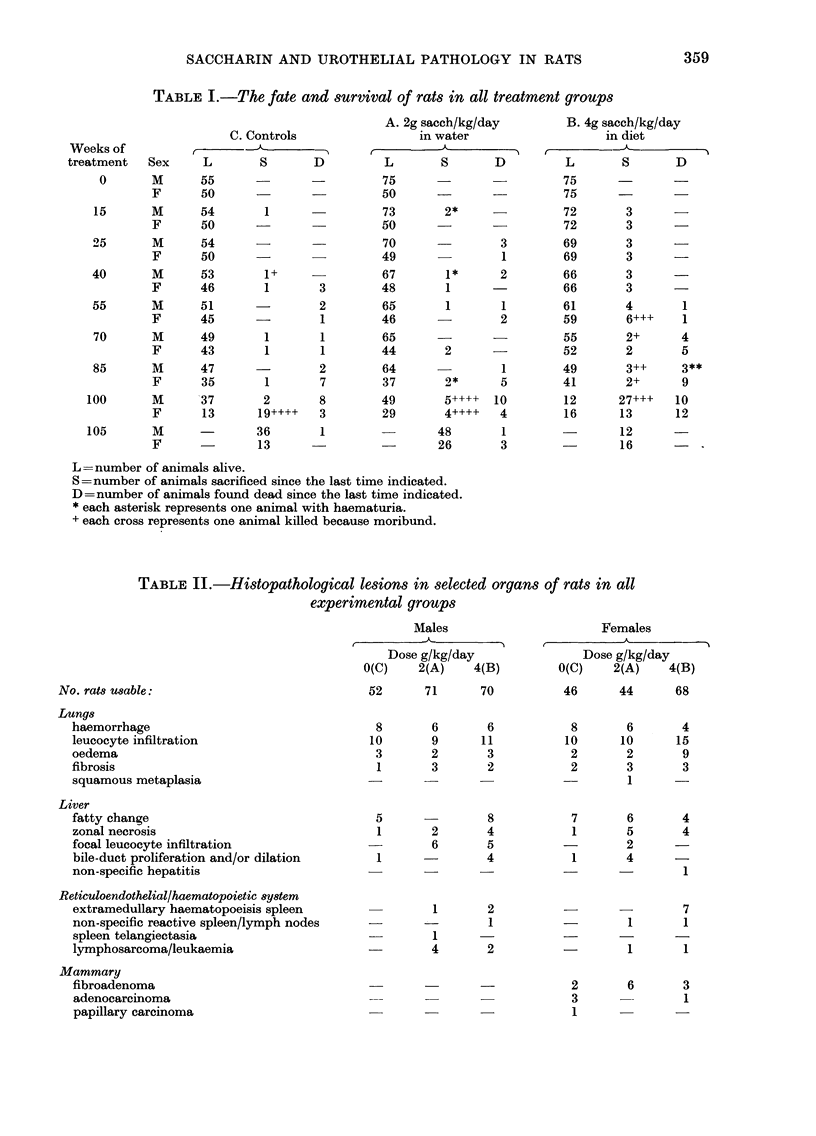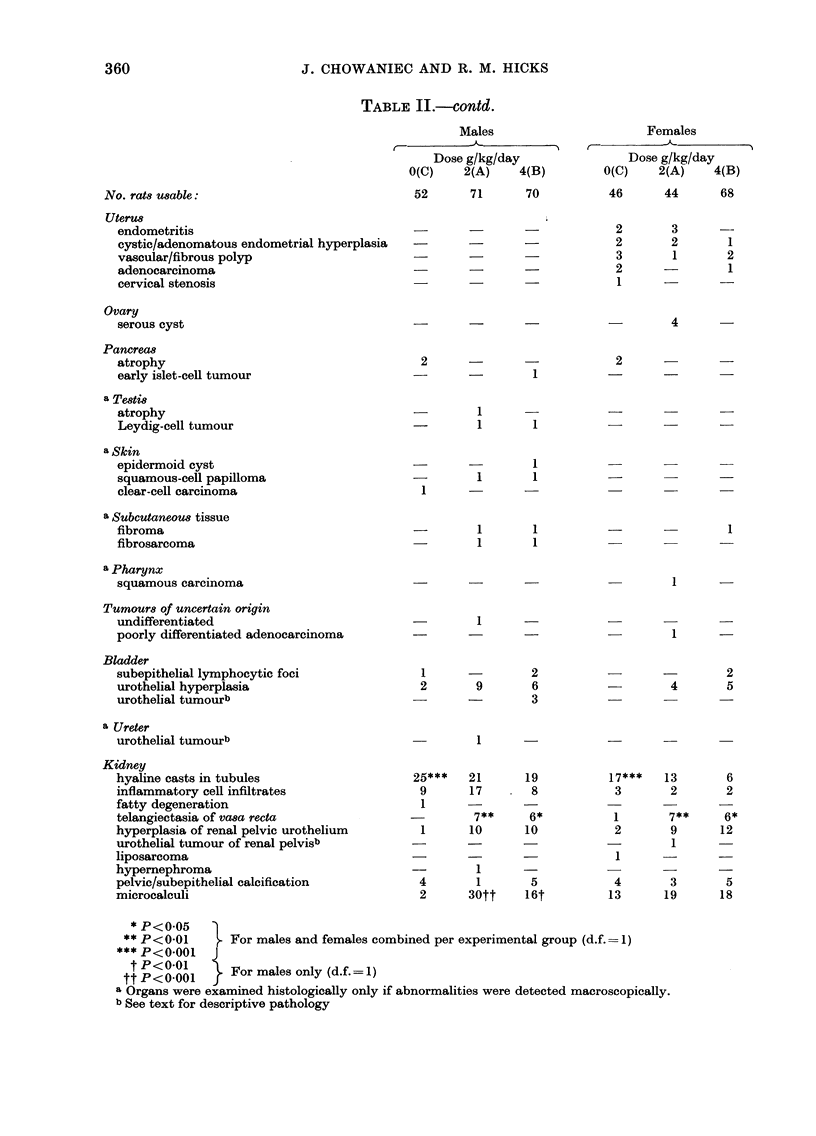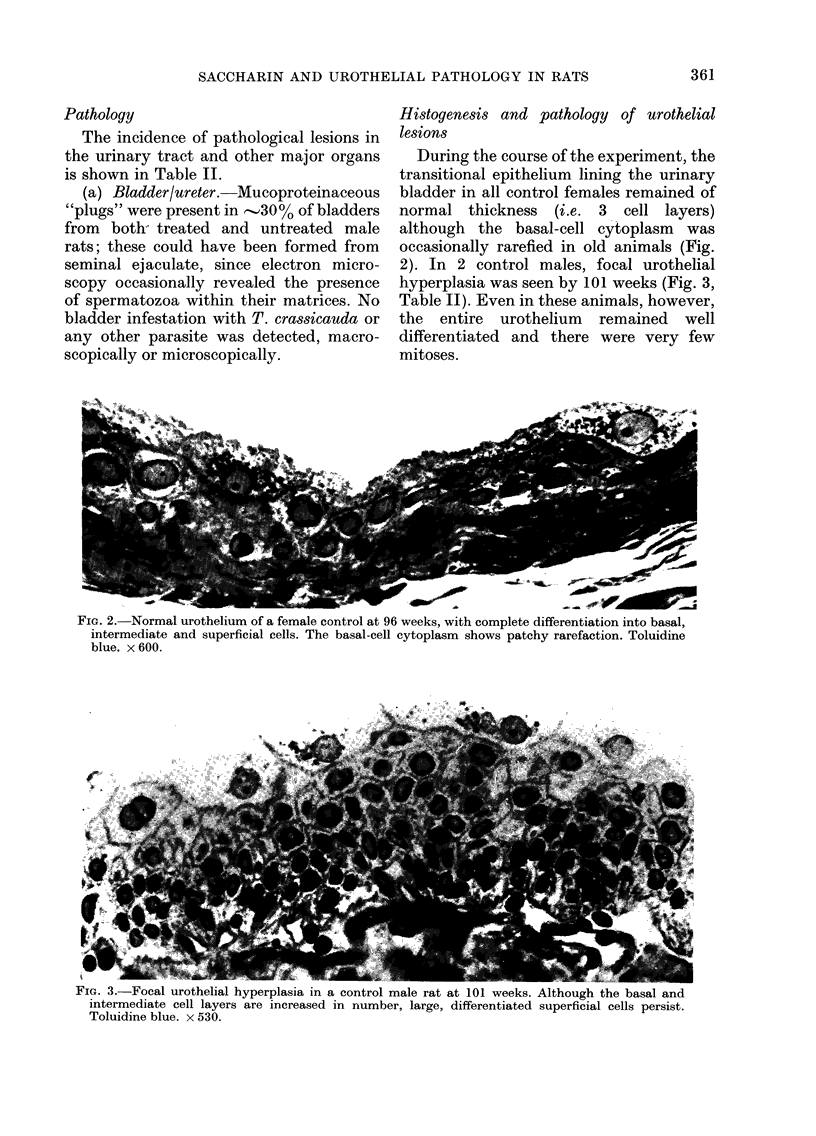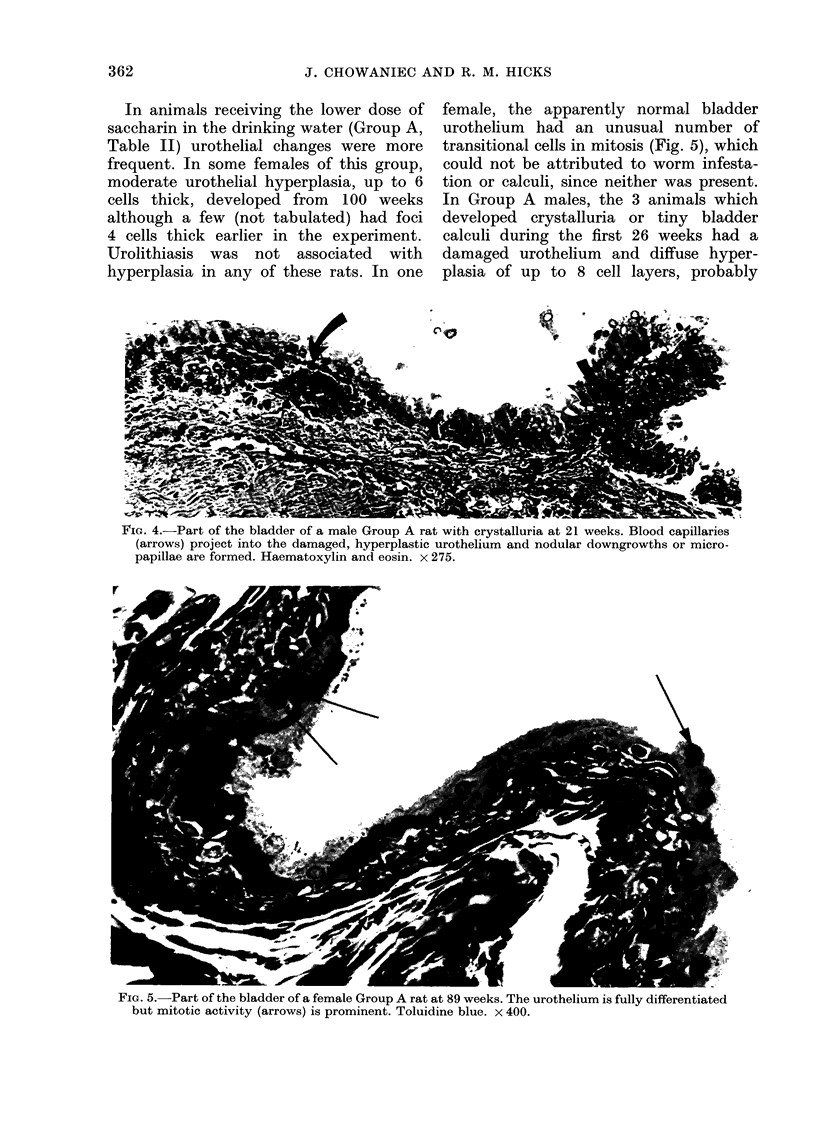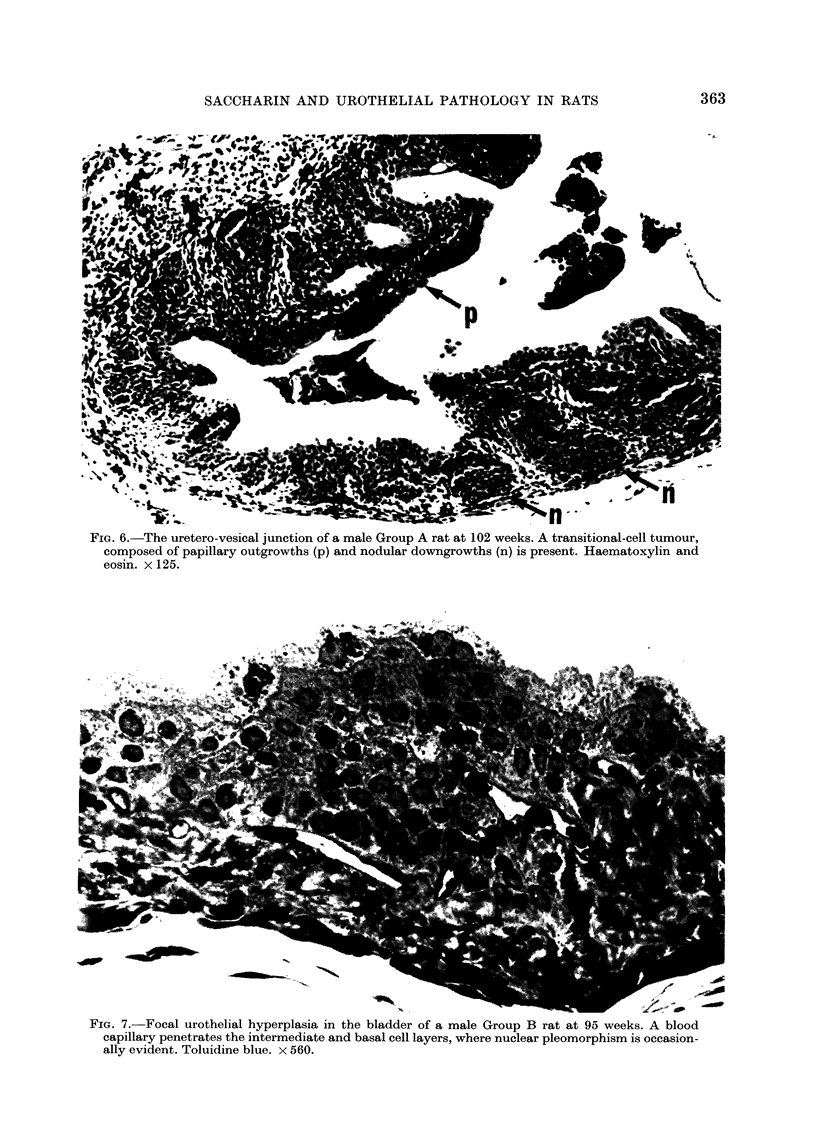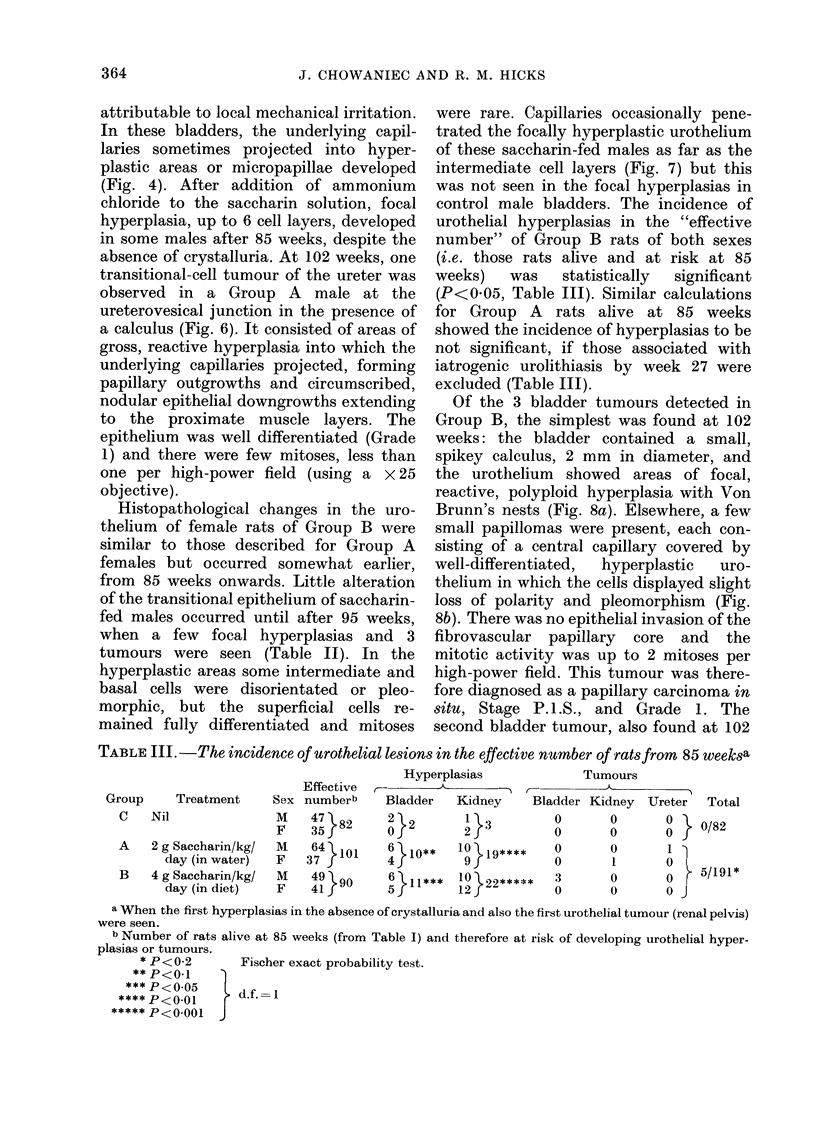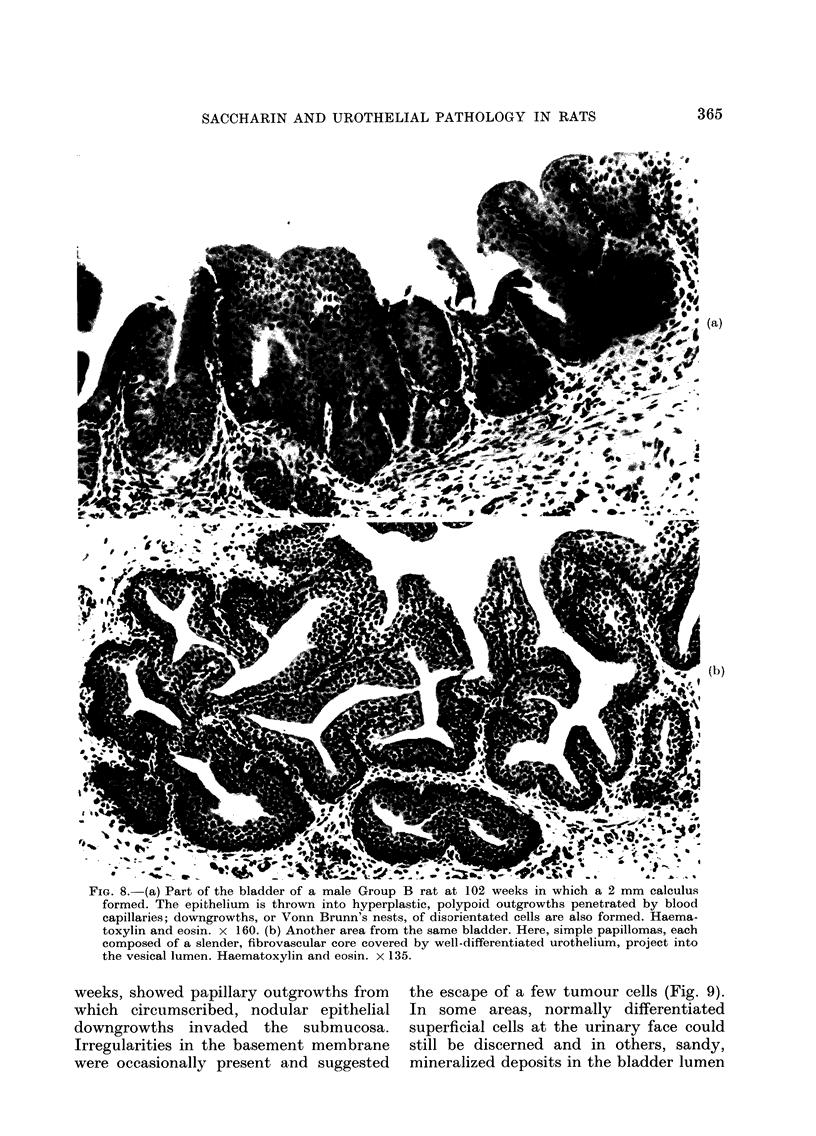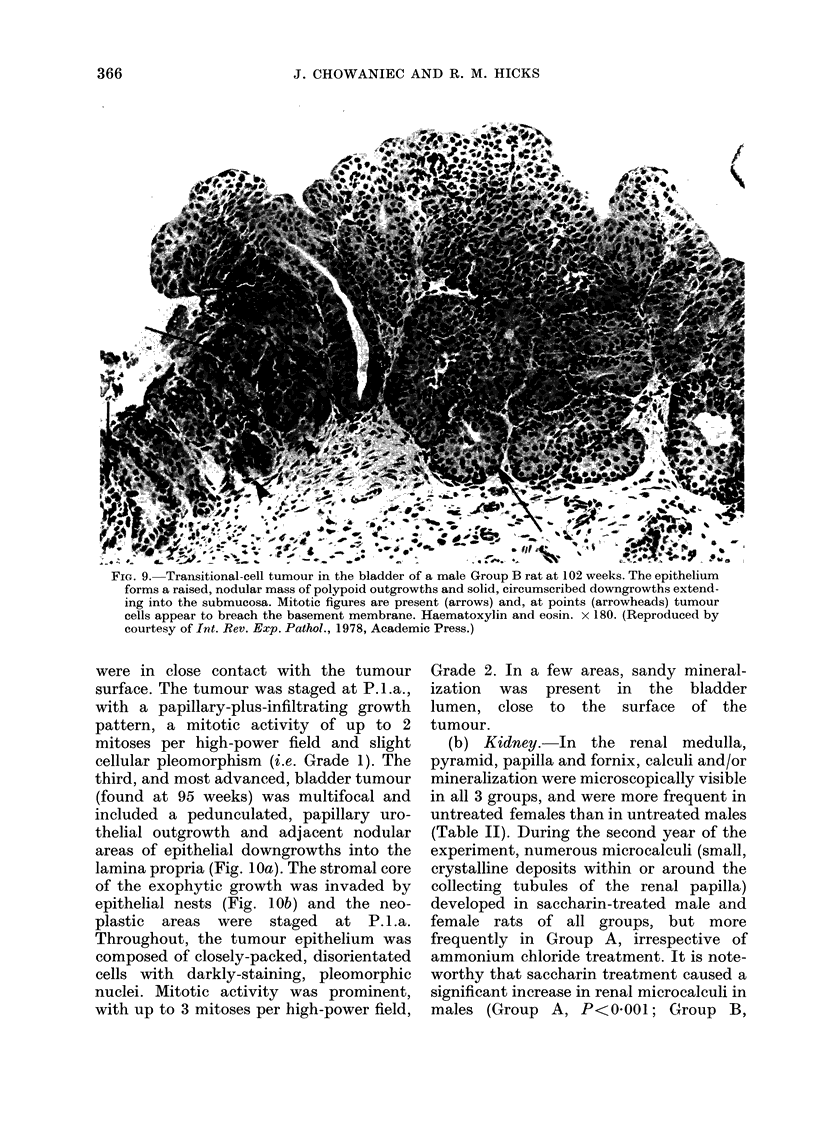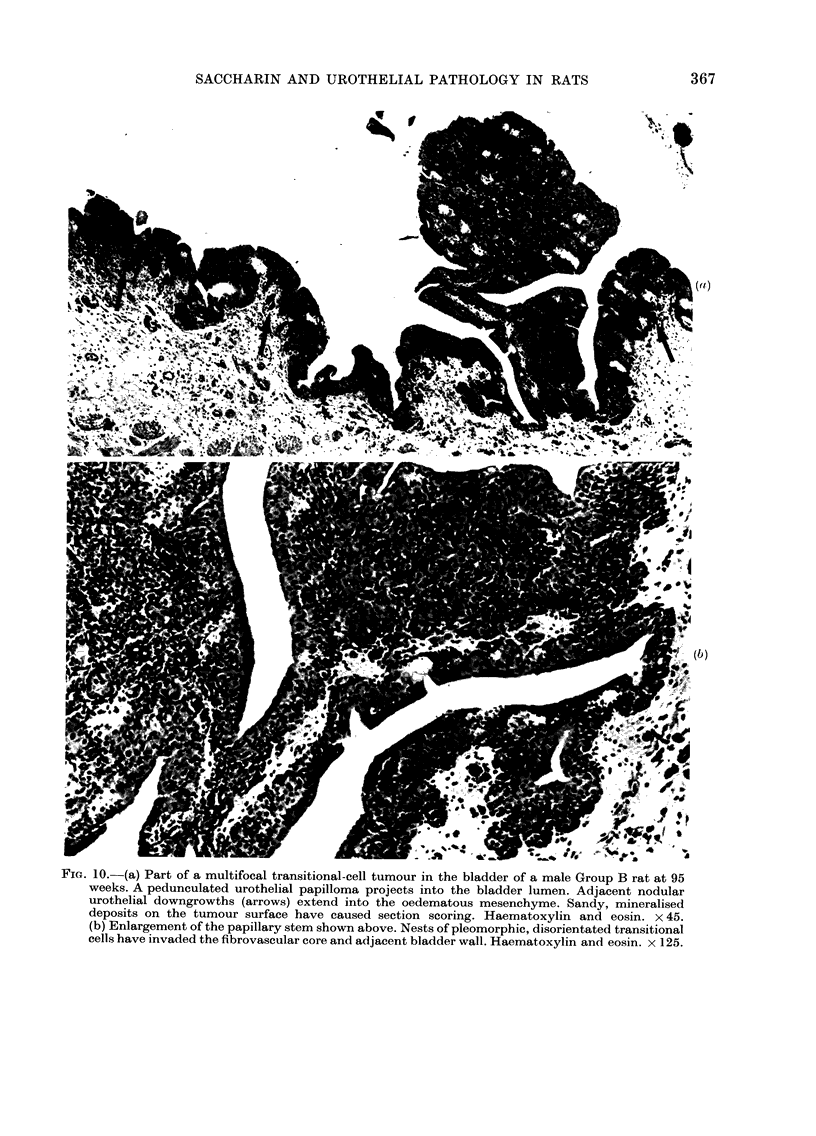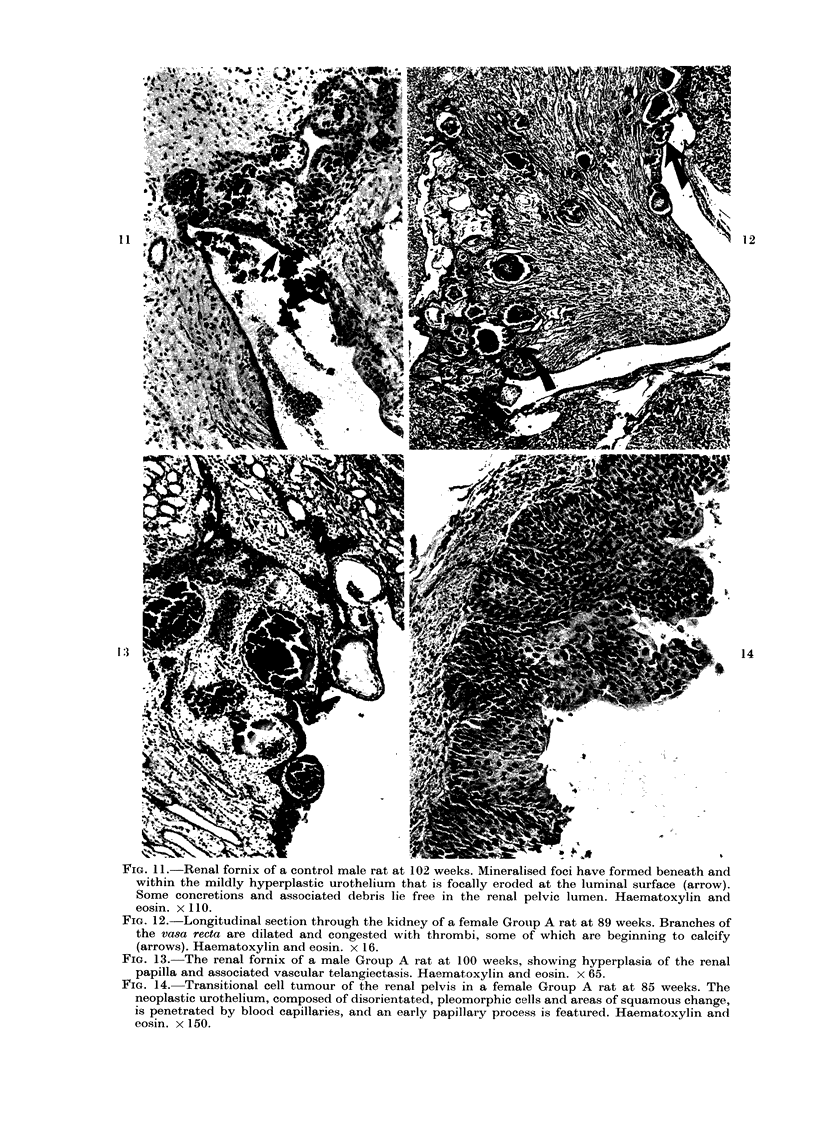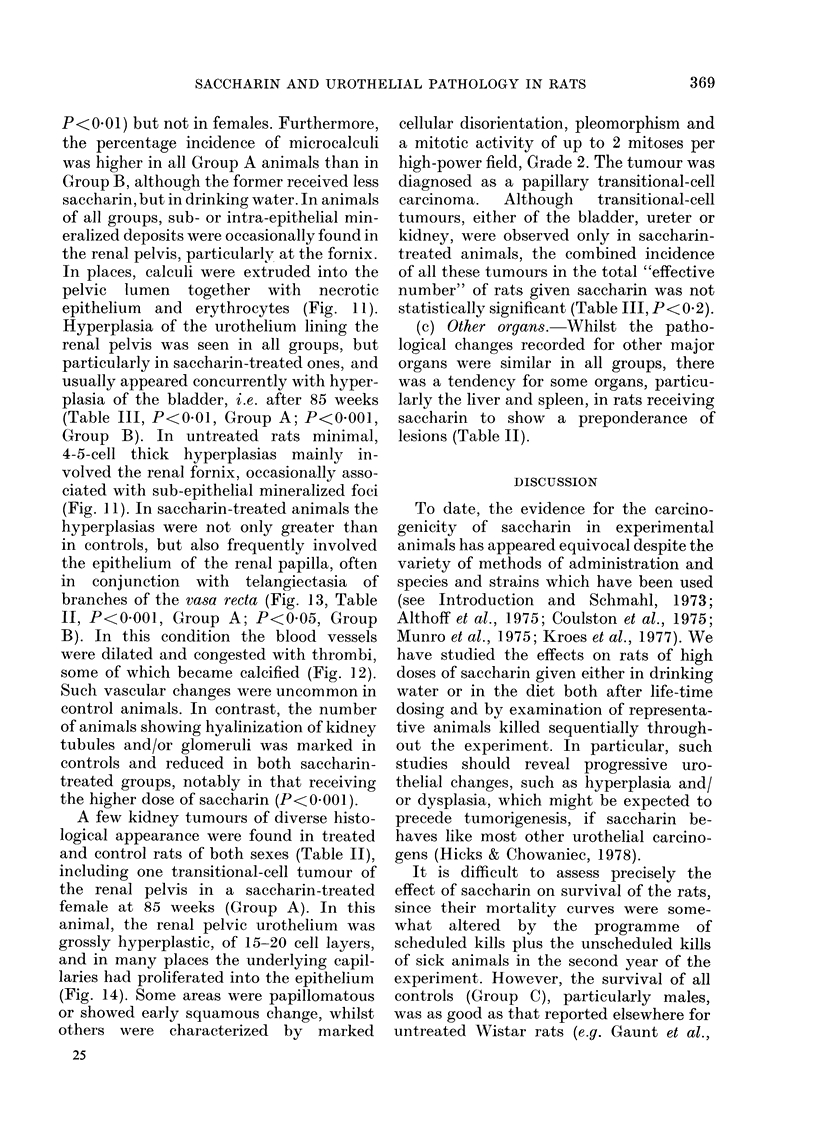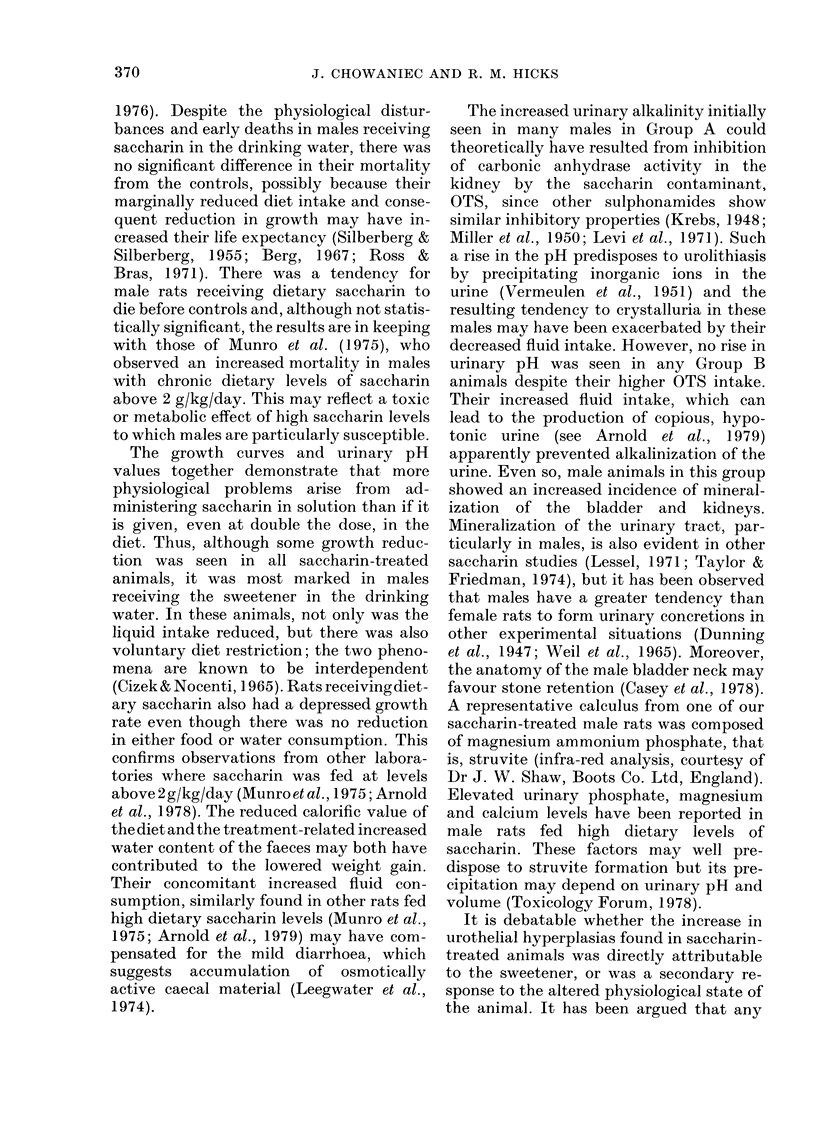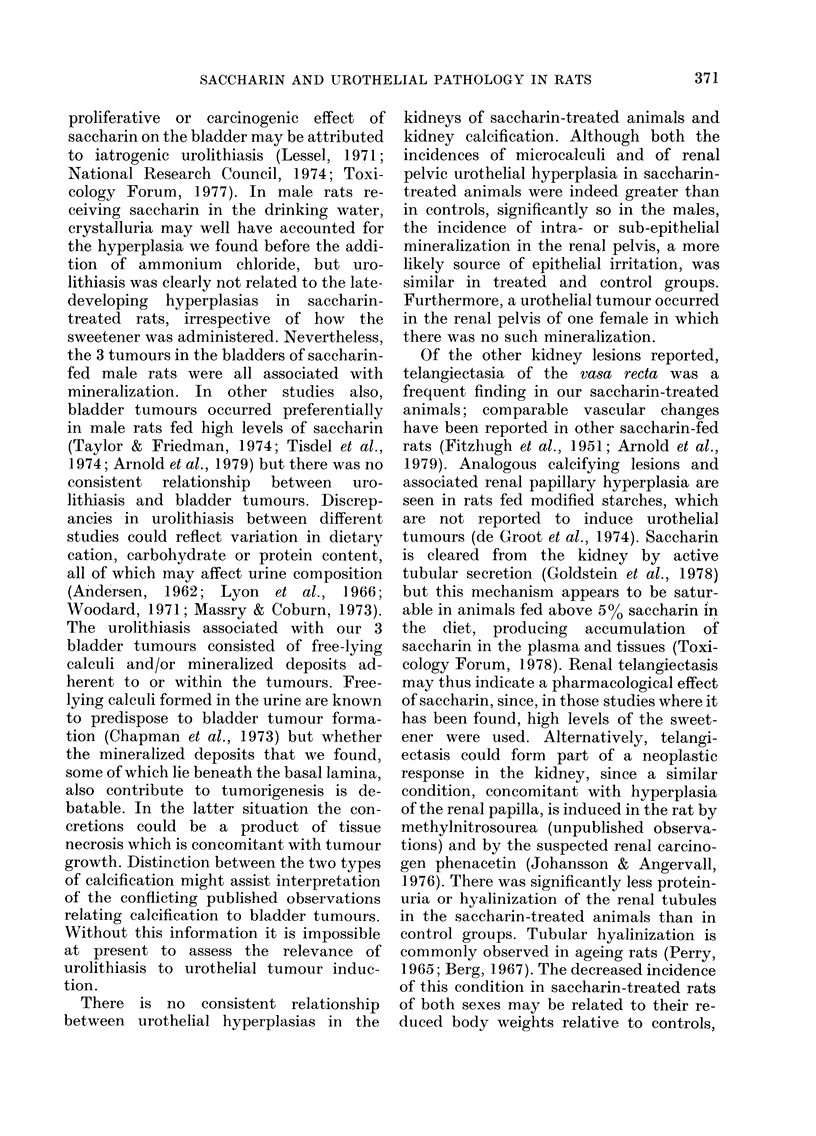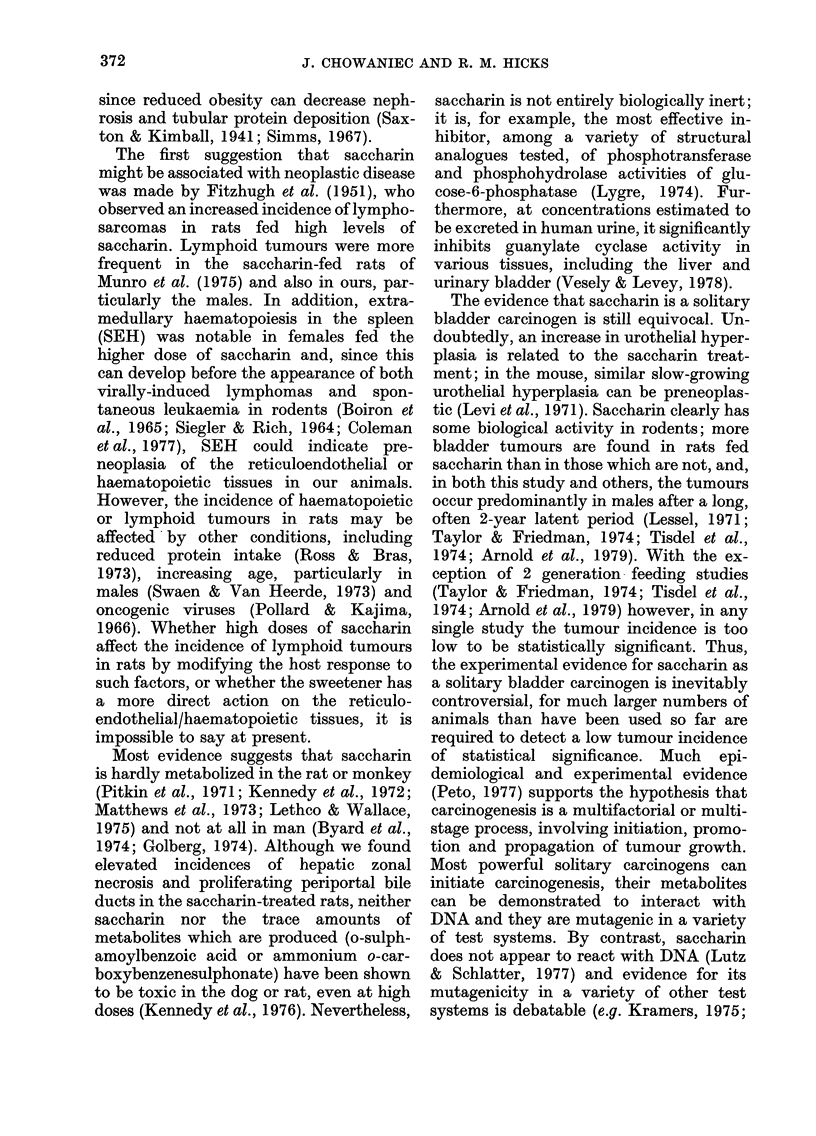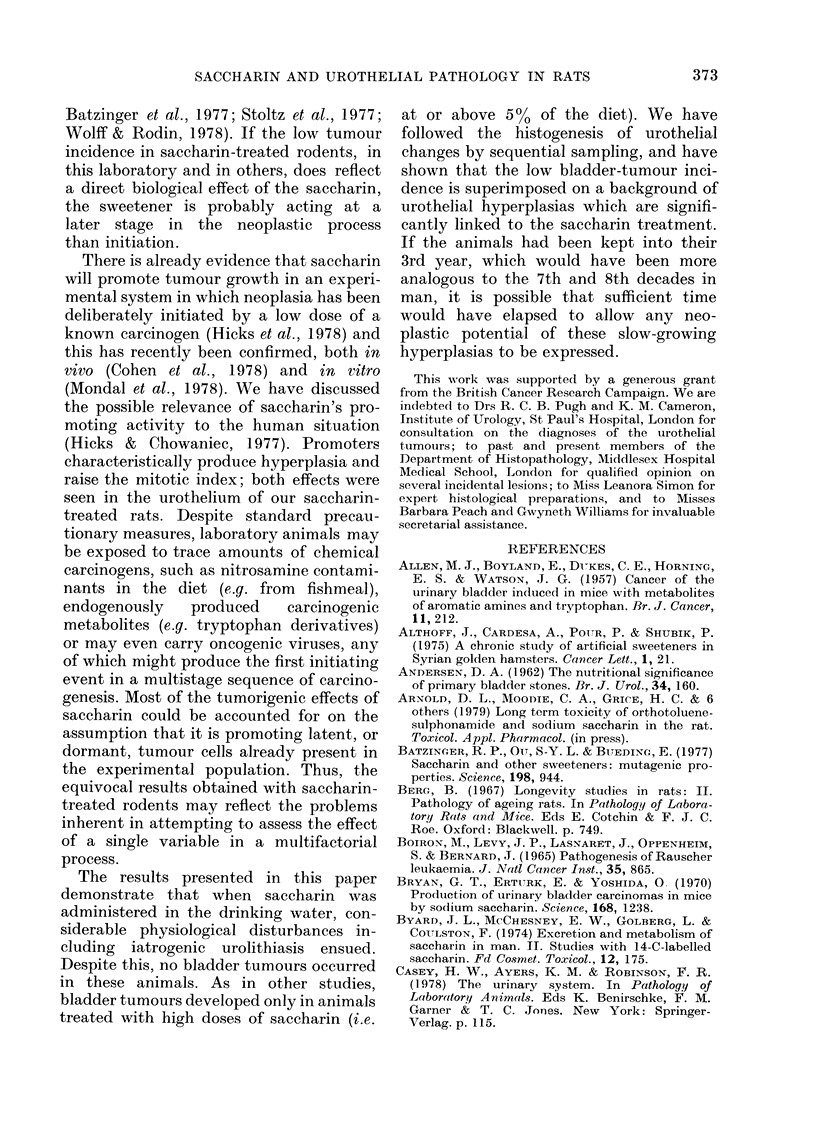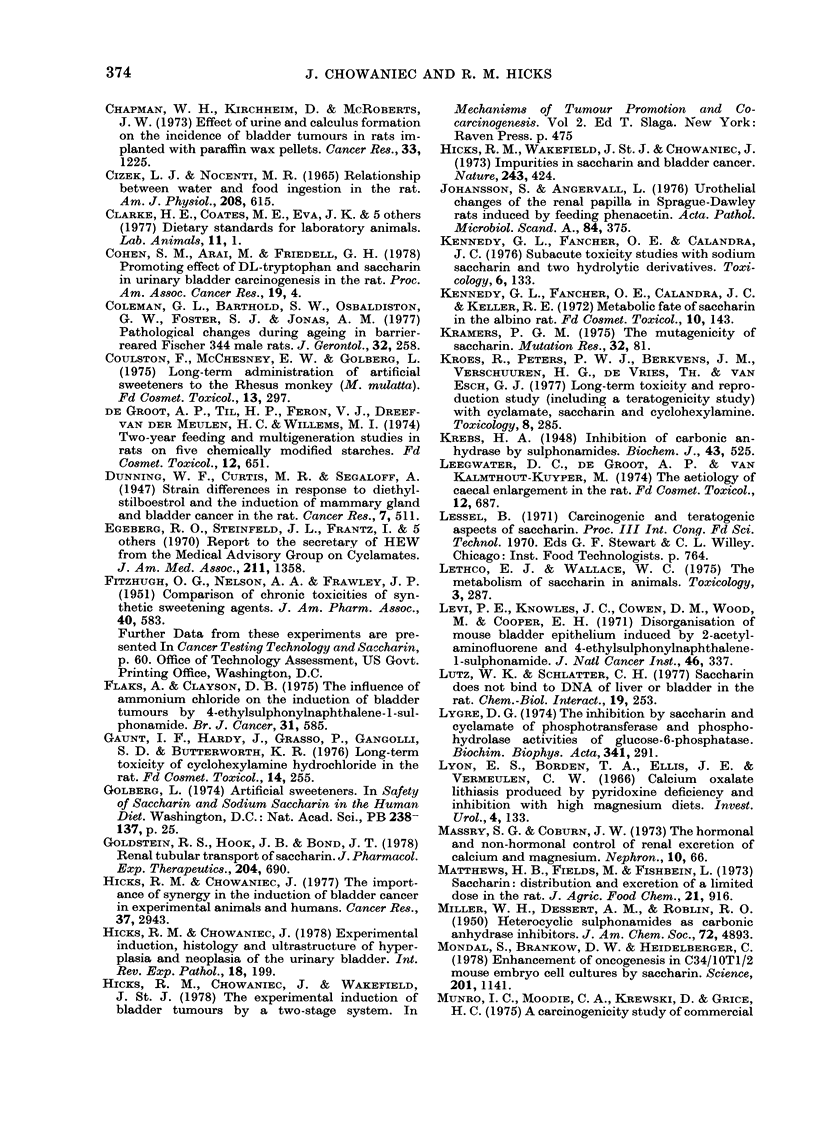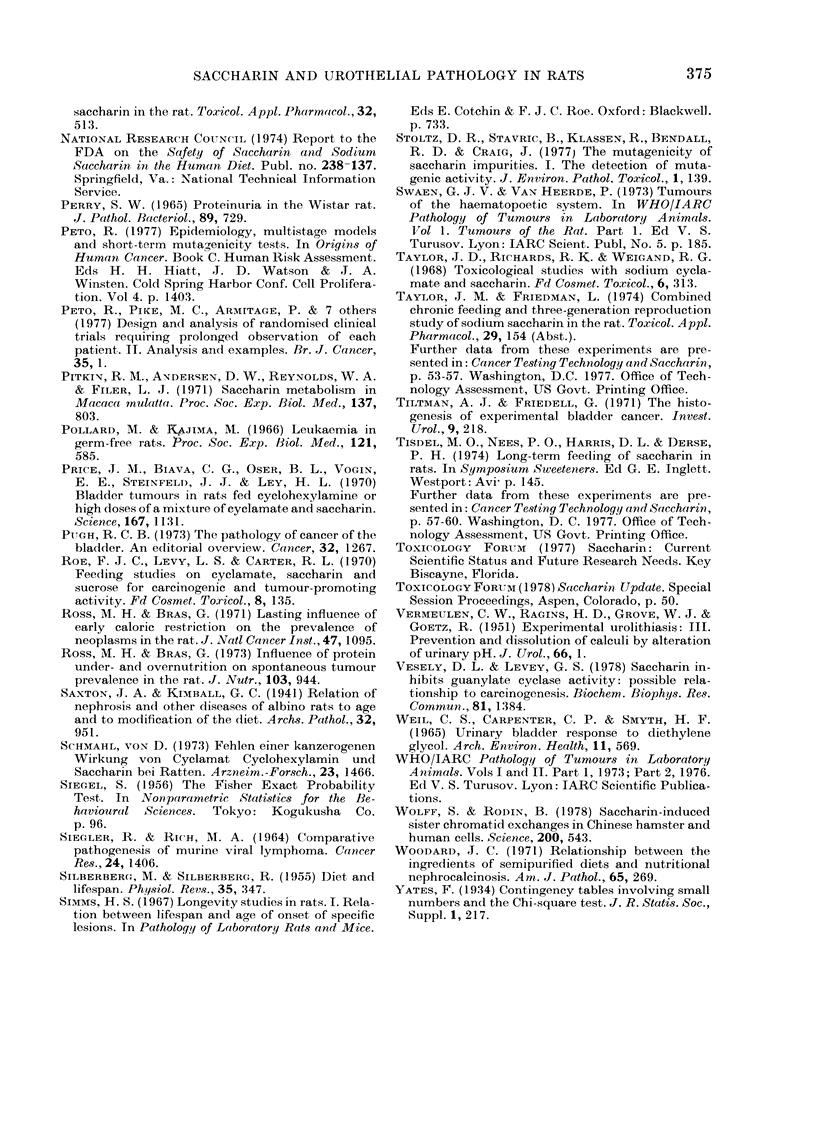# Response of the rat to saccharin with particular reference to the urinary bladder.

**DOI:** 10.1038/bjc.1979.68

**Published:** 1979-04

**Authors:** J. Chowaniec, R. M. Hicks

## Abstract

**Images:**


Br. J. Cancer (1979) 39, 355

### RESPONSE OF THE RAT TO SACCHARIN WITH PARTICULAR REFERENCE TO THE URINARY BLADDER

J. CHOWANIEC AND R. M. HICKS

*From the School of Pathology, Middlesex Hospital Medical School, London*

Received 15 November 1978 Accepted 8 January 1979

**Summary.—Male and female Wistar rats were administered sodium saccharin for life (2 yr) either in the drinking water or diet. The maximum palatable dose of saccharin in the drinking water was found to be 2 g/kg/day and, even then, there was some voluntary restriction of fluid intake in the males. By contrast, double this dose —namely 4 g/kg/day, was palatable in the diet. A control group of rats of both sexes received saccharin-free diet and drinking water. Mild urothelial hyperplasias developed from 85 weeks in rats of both sexes receiving saccharin either in the drinking water or diet; the incidence was statistically significant in both the bladders and kidneys of rats receiving the higher dose of saccharin in the diet, but in the kidneys only of rats receiving the lower dose of saccharin in the drinking water. Telangiectasia of the *vasa recta* was significant in saccharin-treated rats of both sexes at both doses. A very low incidence of bladder tumours, exclusively in males receiving the higher saccharin dose in the diet was seen from 95 weeks. No consistent relationship between bladder epithelial hyperplasias and crystalluria could be demonstrated, although all 3 bladder tumours were associated with some form of mineralisation. Results suggest a particular susceptibility of males to saccharin treatment. The possibility that saccharin may promote, or enhance, the development of latent tumour cells already present in the experimental population, rather than initiate carcinogenesis *per se* is considered.**

SACCHARIN came under suspicion as a possible bladder carcinogen after the withdrawal of cyclamate from the GRAS (generally regarded as safe) list in 1969 (Egeberg *et al.*, 1970), following preliminary reports that a dose-related incidence of urinary-bladder tumours developed in rats fed high doses of a 10:1 cyclamate: saccharin mixture (Price *et al.*, 1970). Although cyclamate was suspected at the time, either saccharin or the combination of both sweeteners could equally well have been responsible for the tumorigenesis. New evidence, subsequently presented by Bryan *et al.* (1970) confirmed earlier observations that implantation of saccharin-containing cholesterol pellets into the urinary bladders of mice increased the bladder-tumour incidence over that induced by implantation of cholesterol pellets alone (Allen *et al.*, 1957). These results conflicted with single-generation feeding studies that had failed to demonstrate a significant carcinogenic effect of saccharin, in the urinary bladder or in any other organ (Fitzhugh *et al.*, 1951; Taylor *et al.*, 1968; Roe *et al.*, 1970; Lessel, 1971).

To attempt to assess what role, if any, saccharin might play in urothelial carcinogenesis, we have investigated the effects of chronic administration to rats of high doses of saccharin, given either in drinking water or in the diet to simulate the normal routes of human exposure. Some of the preliminary, numerical data from these experiments have been published (Hicks & Chowaniec, 1977; Hicks *et al.*, 1978). In common with concurrent trials in different laboratories (Taylor & Friedman, 1974; Tisdel *et al.*, 1974; Arnold *et al.*, 1979) we observed a low incidence of bladder tumours in saccharin-fed male animals. The bladder pathology is discussed in relation to the degree of urolithiasis observed, which differed according to the method of saccharin administration. This paper describes histopathological changes in the urinary bladder throughout the animals' life-span and also some incidental pathological effects observed in other organs. Our results are related to those of recent saccharin studies in other laboratories, and the possible mechanisms of action of saccharin on the urothelium are discussed.

### MATERIALS AND METHODS

*The sodium saccharin* (supplied by Fisons, Loughborough, Leicestershire; manufactured by Boots Co., Nottingham, England) was a fine, white crystalline powder, soluble in water. Gas-liquid chromatographic analysis (by courtesy of Dr B. Stavric, Bureau of Chemical Safety, Health Protection Branch, Health and Welfare Canada, Ottawa) showed it to contain small amounts of several organic solvent-soluble impurities, notably 5 isomers of ditolylsulphone. Quantitative analysis demonstrated the major impurity to be orthotoluenesulphonamide (OTS), for which repeated measurements gave an average level of 698 parts/$10^6$ (B. Stavric, personal communication). A similar result was obtained by the Battelle Institute from a single sample of this saccharin (Hicks *et al.*, 1973).

*Animals and diet.*—Specific-pathogen-free Wistar rats, about 8 weeks old at the start of the experiment, were maintained on pencilled, Standard 41B Laboratory Rat Diet (E. Dixon and Co., Ware, Herts, England) the composition and pesticide-residue levels of which have been outlined by Clarke *et al.* (1977). The standard vitamin mix contained 8000 i.u. vitamin A/kg of diet and the mineral mix contained, in addition to NaCl, 44·50 parts/$10^6$ Fe, 2·54 parts/$10^6$ Cu, 2·00 parts/$10^6$ I, 74·00 parts/$10^6$ Mn, 0·64 parts/$10^6$ Co and 91·50 parts/$10^6$ Mg. No detectable levels of aflatoxin were present. Tap water was provided for all animals. Saccharin was incorporated either into the diet or drinking water for some groups of animals as indicated below. Diet and drinking water were available *ad libitum*. The experiment was terminated at 2 years.

*Experimental groups.*—The rats were divided randomly into 3 experimental groups, 2 of which were administered sodium saccharin orally.

Group A. Saccharin in drinking water: 75 male and 50 female rats were given the sodium saccharin in the drinking water. The concentration of this solution was periodically adjusted so that the average dose received was 2 g saccharin/kg/day (equivalent to about 4% of the diet). Higher doses were unpalatable and the animals would not drink sufficient to remain healthy.

Group B. Saccharin in the diet: 75 males and 75 females received the sodium saccharin incorporated into pencilled, 41B diet (courtesy of E. Dixon and Co., Ware, Herts, England) to give an average dose of 4 g/kg/day (7–8% of the diet), which was well tolerated.

Group C. Controls: 55 male and 50 female rats remained untreated.

*Animal maintenance.*—The rats were housed in groups of 5 in air-filtered rooms at 19–22°C with a relative humidity of 55–60%. Their weights, food and water intakes were measured at regular intervals. Changes in the quality of fur, eyes, general physical appearance or behaviour and the development of haematuria or palpable masses were recorded. Sick or infected animals were immediately isolated. Urinary pH was measured periodically and, because an increased urine alkalinity and urolithiasis was observed in some Group A males by 27 weeks, all males in this group were then given sodium saccharin in a 1% solution of ammonium chloride to restore urine acidity (Levi *et al.*, 1971; Flaks & Clayson, 1975). This saccharin/ammonium chloride mixture was obviously distasteful and, after 4 weeks, the ammonium chloride concentration was therefore reduced to 0·5% and maintained at this level for all Group A males for the duration of the experiment. Twenty-five of the control males (Group C) were given ammonium chloride at the same concentrations. No differences in weight, food or fluid consumption were observed between these animals and the untreated male controls. No similar urinary pH problems were encountered in females receiving saccharin in the drinking water or in animals of either sex receiving the higher dose of saccharin in the diet.

*Sampling of animals for pathological changes.*—To assess any treatment-related morphological alterations of the bladder epithelium, rats from all groups were periodically sacrificed. Animals were chosen at random unless any individuals presented with haematuria, or were found moribund, in which case these were preferentially selected (Table I). Throughout both the first and second years all animals found dead were autopsied and, where possible, their major organs processed for histology. Survivors were killed by cervical dislocation between weeks 94 and 105. The urethra was clamped and the bladder transmurally inflated with 0·5–1·0 ml of 0·1M cacodylate-buffered 4% formaldehyde (pH 7·4) for 5 min. The bladder was then excised, bisected longitudinally and transilluminated with a dissecting microscope. This procedure allowed calculi or tumours as small as 1·0 mm diam. to be detected, and the bladders were also carefully inspected for the presence of parasites. Representative areas from apparently normal and abnormal bladders were processed for electron microscopy (report in preparation) and the remainder of each bladder was fixed in the cacodylate-buffered formaldehyde. All major organs were surveyed macroscopically. Both kidneys and samples of lung, liver, spleen, pancreas, ovaries and uterus were similarly fixed for histopathological assessment. Any other organ showing an abnormality was also taken. Sections of the paraffin-embedded tissues were stained with Cole's haematoxylin and eosin.

*Histopathological assessment of tumours and statistical analysis.*—The WHO classification outlined by Pugh (1973) was used to grade and stage the bladder tumours. Patterns of bladder tumour growth were also assessed according to Tiltman & Friedell (1971). Incidental pathology was diagnosed using WHO/IARC criteria for the rat (1973; 1976). Throughout, the data were statistically analysed using the Chi-square test for $2 \times 2$ contingency tables with a correction for small numbers according to Yates (1934). However, where the smallest expected value was less than 5, this test was inappropriate and the Fisher Exact Probability Test (Siegel, 1956) was employed instead, where indicated in the Results. For statistical analysis of the incidence of urothelial lesions, the "effective number" of rats, defined as the number of rats from each experimental group considered to have lived long enough for a particular urothelial lesion to develop, was used. The length of time necessary for development, or "effective time", was the period which elapsed before observation of the first lesion in that group. Hence, if the first urothelial lesion, *e.g.* hyperplasia, was observed at X weeks, the number of rats alive at X weeks was considered the "effective number" and X weeks the "effective time". This eliminated from the calculations those animals which had died or were sacrificed before the latent period of hyperplasia formation had elapsed, leaving only the true number at risk.

### RESULTS

### *Urine pH and the development of crystalluria*

Throughout the 2 yr experiment the urine pH value of untreated control rats (Group C), both male and female, averaged between 6·0 and 6·5. Similar values were recorded for both Group B (4 g saccharin/kg/day in the diet) males and females and Group A (2 g saccharin/kg/day in water) females. In Group A males, however, by 27 weeks the average urinary pH had risen to above 7·0 and some individuals within this group had pH values of 8·5 or 9·0. In 3 of these animals, the rise in pH was accompanied by marked crystalluria, sufficient to cause urethral blockage and subsequent death. To prevent further mortality, the pH changes in Group A males were corrected by addition of ammonium chloride to the saccharin in the drinking water, thus returning their urinary pH to 6·0 or 6·5. No gross crystalluria was subsequently found but the ammonium chloride did not prevent development of microcalculi in their kidneys, the percentage incidence of which was similar to that in Group A females not receiving ammonium chloride (see Kidney Pathology).

In the bladders of 3 saccharin-fed males from Group B, mineralized deposits or free-lying calculi were present; the 2 largest calculi measured 2 mm in diameter. The mineralized deposits were detected microscopically and consisted of crystalline material adherent to, or within the urothelium or mucoproteinaceous secretions.

FIG. 1.—Weight gain of rats in the 3 treatment groups from Week 0 to Week 80 of the experiment. A. Males. B. Females.
●——● untreated controls.
■——■ 2 g saccharin/kg/day (in water).
▲——▲ 4 g saccharin/kg/day (in diet).

*Growth*

(1) Group A animals (saccharin 2 g/kg/day, in drinking water). This intake of saccharin was associated with a slower weight gain and a final body weight lower than controls for both sexes (Fig. 1). Liquid intake decreased early in the experiment, especially in males. After addition of ammonium chloride to their drinking water, there was an increased fluid consumption and their liquid intake returned to 90% of control values. In females also, liquid consumption remained slightly below that of controls. Food consumption in saccharin-treated males was reduced by 10% of control values but in females, remained comparable to that of controls.

(2) Group B animals (saccharin 4 g/kg/day, in the diet). The weight gain for males in Group B was greater than for those of Group A, but final body weights were still lower than controls for both sexes (Fig. 1). The depression in growth was not, however, accompanied by reduced food or water intakes for either sex. Indeed, during the second year, fluid consumption rose by an average of 100% in males and 35% in females above control values. Stool-softening or mild diarrhoea also occurred.

*Survival*

The numbers of rats alive at, and those which died or were sacrificed between, defined times in all experimental groups are presented in Table I. By 85 weeks mortality was generally higher among all females than males, but the difference was not statistically significant in any group. Despite the initial rise in mortality due to urolithiasis in Group A males, the overall mortality rate in this group did not significantly differ from controls. A marginal increase in death rate was apparent towards the end of the experiment in Group B males. However, at the 5% significance level, there was no difference in survival or extent of exposure to risk of death (Peto *et al.*, 1977) between any treated and/or untreated group for either sex at any time considered, regardless of whether the randomly sacrificed animals were included or excluded from the calculations, as estimated from Table I.

TABLE I.—*The fate and survival of rats in all treatment groups*

| Weeks of treatment | Sex | C. Controls L | S | D | A. 2g sacch/kg/day in water L | S | D | B. 4g sacch/kg/day in diet L | S | D |
|---|---|---|---|---|---|---|---|---|---|---|
| 0 | M | 55 | — | — | 75 | — | — | 75 | — | — |
| | F | 50 | — | — | 50 | — | — | 75 | — | — |
| 15 | M | 54 | 1 | — | 73 | 2* | — | 72 | 3 | — |
| | F | 50 | — | — | 50 | — | — | 72 | 3 | — |
| 25 | M | 54 | — | — | 70 | — | 3 | 69 | 3 | — |
| | F | 50 | — | — | 49 | — | 1 | 69 | 3 | — |
| 40 | M | 53 | $1^{+}$ | — | 67 | 1* | 2 | 66 | 3 | — |
| | F | 46 | 1 | 3 | 48 | 1 | — | 66 | 3 | — |
| 55 | M | 51 | — | 2 | 65 | 1 | 1 | 61 | 4 | 1 |
| | F | 45 | — | 1 | 46 | — | 2 | 59 | $6^{+++}$ | 1 |
| 70 | M | 49 | 1 | 1 | 65 | — | — | 55 | $2^{+}$ | 4 |
| | F | 43 | 1 | 1 | 44 | 2 | — | 52 | 2 | 5 |
| 85 | M | 47 | — | 2 | 64 | — | 1 | 49 | $3^{++}$ | 3** |
| | F | 35 | 1 | 7 | 37 | 2* | 5 | 41 | $2^{+}$ | 9 |
| 100 | M | 37 | 2 | 8 | 49 | $5^{++++}$ | 10 | 12 | $27^{+++}$ | 10 |
| | F | 13 | $19^{++++}$ | 3 | 29 | $4^{++++}$ | 4 | 16 | 13 | 12 |
| 105 | M | — | 36 | 1 | — | 48 | 1 | — | 12 | — |
| | F | — | 13 | — | — | 26 | 3 | — | 16 | — |

L = number of animals alive.
S = number of animals sacrificed since the last time indicated.
D = number of animals found dead since the last time indicated.
* each asterisk represents one animal with haematuria.
$^{+}$ each cross represents one animal killed because moribund.

TABLE II.—*Histopathological lesions in selected organs of rats in all experimental groups*

| | Males Dose g/kg/day 0(C) | 2(A) | 4(B) | Females Dose g/kg/day 0(C) | 2(A) | 4(B) |
|---|---|---|---|---|---|---|
| *No. rats usable:* | 52 | 71 | 70 | 46 | 44 | 68 |
| *Lungs* | | | | | | |
| haemorrhage | 8 | 6 | 6 | 8 | 6 | 4 |
| leucocyte infiltration | 10 | 9 | 11 | 10 | 10 | 15 |
| oedema | 3 | 2 | 3 | 2 | 2 | 9 |
| fibrosis | 1 | 3 | 2 | 2 | 3 | 3 |
| squamous metaplasia | — | — | — | — | 1 | — |
| *Liver* | | | | | | |
| fatty change | 5 | — | 8 | 7 | 6 | 4 |
| zonal necrosis | 1 | 2 | 4 | 1 | 5 | 4 |
| focal leucocyte infiltration | — | 6 | 5 | — | 2 | — |
| bile-duct proliferation and/or dilation | 1 | — | 4 | 1 | 4 | — |
| non-specific hepatitis | — | — | — | — | — | 1 |
| *Reticuloendothelial/haematopoietic system* | | | | | | |
| extramedullary haematopoeisis spleen | — | 1 | 2 | — | — | 7 |
| non-specific reactive spleen/lymph nodes | — | — | 1 | — | 1 | 1 |
| spleen telangiectasia | — | 1 | — | — | — | — |
| lymphosarcoma/leukaemia | — | 4 | 2 | — | 1 | 1 |
| *Mammary* | | | | | | |
| fibroadenoma | — | — | — | 2 | 6 | 3 |
| adenocarcinoma | — | — | — | 3 | — | 1 |
| papillary carcinoma | — | — | — | 1 | — | — |

TABLE II.—*contd.*

| | Males | | | Females | | |
|---|---|---|---|---|---|---|
| | Dose g/kg/day | | | Dose g/kg/day | | |
| | 0(C) | 2(A) | 4(B) | 0(C) | 2(A) | 4(B) |
| *No. rats usable:* | 52 | 71 | 70 | 46 | 44 | 68 |
| *Uterus* | | | | | | |
| endometritis | — | — | — | 2 | 3 | — |
| cystic/adenomatous endometrial hyperplasia | — | — | — | 2 | 2 | 1 |
| vascular/fibrous polyp | — | — | — | 3 | 1 | 2 |
| adenocarcinoma | — | — | — | 2 | — | 1 |
| cervical stenosis | — | — | — | 1 | — | — |
| *Ovary* | | | | | | |
| serous cyst | — | — | — | — | 4 | — |
| *Pancreas* | | | | | | |
| atrophy | 2 | — | — | 2 | — | — |
| early islet-cell tumour | — | — | 1 | — | — | — |
| [a] *Testis* | | | | | | |
| atrophy | — | 1 | — | — | — | — |
| Leydig-cell tumour | — | 1 | 1 | — | — | — |
| [a] *Skin* | | | | | | |
| epidermoid cyst | — | — | 1 | — | — | — |
| squamous-cell papilloma | — | 1 | 1 | — | — | — |
| clear-cell carcinoma | 1 | — | — | — | — | — |
| [a] *Subcutaneous* tissue | | | | | | |
| fibroma | — | 1 | 1 | — | — | 1 |
| fibrosarcoma | — | 1 | 1 | — | — | — |
| [a] *Pharynx* | | | | | | |
| squamous carcinoma | — | — | — | — | 1 | — |
| *Tumours of uncertain origin* | | | | | | |
| undifferentiated | — | 1 | — | — | — | — |
| poorly differentiated adenocarcinoma | — | — | — | — | 1 | — |
| *Bladder* | | | | | | |
| subepithelial lymphocytic foci | 1 | — | 2 | — | — | 2 |
| urothelial hyperplasia | 2 | 9 | 6 | — | 4 | 5 |
| urothelial tumour[b] | — | — | 3 | — | — | — |
| [a] *Ureter* | | | | | | |
| urothelial tumour[b] | — | 1 | — | — | — | — |
| *Kidney* | | | | | | |
| hyaline casts in tubules | 25*** | 21 | 19 | 17*** | 13 | 6 |
| inflammatory cell infiltrates | 9 | 17 | 8 | 3 | 2 | 2 |
| fatty degeneration | 1 | — | — | — | — | — |
| telangiectasia of *vasa recta* | — | 7** | 6* | 1 | 7** | 6* |
| hyperplasia of renal pelvic urothelium | 1 | 10 | 10 | 2 | 9 | 12 |
| urothelial tumour of renal pelvis[b] | — | — | — | — | 1 | — |
| liposarcoma | — | — | — | 1 | — | — |
| hypernephroma | — | 1 | — | — | — | — |
| pelvic/subepithelial calcification | 4 | 1 | 5 | 4 | 3 | 5 |
| microcalculi | 2 | 30†† | 16† | 13 | 19 | 18 |

* $P<0.05$, ** $P<0.01$, *** $P<0.001$ } For males and females combined per experimental group (d.f. = 1)

† $P<0.01$, †† $P<0.001$ } For males only (d.f. = 1)

[a] Organs were examined histologically only if abnormalities were detected macroscopically.
[b] See text for descriptive pathology

*Pathology*

The incidence of pathological lesions in the urinary tract and other major organs is shown in Table II.

(a) *Bladder/ureter.*—Mucoproteinaceous "plugs" were present in ~30% of bladders from both treated and untreated male rats; these could have been formed from seminal ejaculate, since electron microscopy occasionally revealed the presence of spermatozoa within their matrices. No bladder infestation with *T. crassicauda* or any other parasite was detected, macroscopically or microscopically.

*Histogenesis and pathology of urothelial lesions*

During the course of the experiment, the transitional epithelium lining the urinary bladder in all control females remained of normal thickness (*i.e.* 3 cell layers) although the basal-cell cytoplasm was occasionally rarefied in old animals (Fig. 2). In 2 control males, focal urothelial hyperplasia was seen by 101 weeks (Fig. 3, Table II). Even in these animals, however, the entire urothelium remained well differentiated and there were very few mitoses.

FIG. 2.—Normal urothelium of a female control at 96 weeks, with complete differentiation into basal, intermediate and superficial cells. The basal-cell cytoplasm shows patchy rarefaction. Toluidine blue. ×600.

FIG. 3.—Focal urothelial hyperplasia in a control male rat at 101 weeks. Although the basal and intermediate cell layers are increased in number, large, differentiated superficial cells persist. Toluidine blue. ×530.

In animals receiving the lower dose of saccharin in the drinking water (Group A, Table II) urothelial changes were more frequent. In some females of this group, moderate urothelial hyperplasia, up to 6 cells thick, developed from 100 weeks although a few (not tabulated) had foci 4 cells thick earlier in the experiment. Urolithiasis was not associated with hyperplasia in any of these rats. In one female, the apparently normal bladder urothelium had an unusual number of transitional cells in mitosis (Fig. 5), which could not be attributed to worm infestation or calculi, since neither was present. In Group A males, the 3 animals which developed crystalluria or tiny bladder calculi during the first 26 weeks had a damaged urothelium and diffuse hyperplasia of up to 8 cell layers, probably

FIG. 4.—Part of the bladder of a male Group A rat with crystalluria at 21 weeks. Blood capillaries (arrows) project into the damaged, hyperplastic urothelium and nodular downgrowths or micropapillae are formed. Haematoxylin and eosin. ×275.

FIG. 5.—Part of the bladder of a female Group A rat at 89 weeks. The urothelium is fully differentiated but mitotic activity (arrows) is prominent. Toluidine blue. ×400.

FIG. 6.—The uretero-vesical junction of a male Group A rat at 102 weeks. A transitional-cell tumour, composed of papillary outgrowths (p) and nodular downgrowths (n) is present. Haematoxylin and eosin. ×125.

FIG. 7.—Focal urothelial hyperplasia in the bladder of a male Group B rat at 95 weeks. A blood capillary penetrates the intermediate and basal cell layers, where nuclear pleomorphism is occasionally evident. Toluidine blue. ×560.

attributable to local mechanical irritation. In these bladders, the underlying capillaries sometimes projected into hyperplastic areas or micropapillae developed (Fig. 4). After addition of ammonium chloride to the saccharin solution, focal hyperplasia, up to 6 cell layers, developed in some males after 85 weeks, despite the absence of crystalluria. At 102 weeks, one transitional-cell tumour of the ureter was observed in a Group A male at the ureterovesical junction in the presence of a calculus (Fig. 6). It consisted of areas of gross, reactive hyperplasia into which the underlying capillaries projected, forming papillary outgrowths and circumscribed, nodular epithelial downgrowths extending to the proximate muscle layers. The epithelium was well differentiated (Grade 1) and there were few mitoses, less than one per high-power field (using a $\times 25$ objective).

Histopathological changes in the urothelium of female rats of Group B were similar to those described for Group A females but occurred somewhat earlier, from 85 weeks onwards. Little alteration of the transitional epithelium of saccharin-fed males occurred until after 95 weeks, when a few focal hyperplasias and 3 tumours were seen (Table II). In the hyperplastic areas some intermediate and basal cells were disorientated or pleomorphic, but the superficial cells remained fully differentiated and mitoses were rare. Capillaries occasionally penetrated the focally hyperplastic urothelium of these saccharin-fed males as far as the intermediate cell layers (Fig. 7) but this was not seen in the focal hyperplasias in control male bladders. The incidence of urothelial hyperplasias in the "effective number" of Group B rats of both sexes (*i.e.* those rats alive and at risk at 85 weeks) was statistically significant ($P<0{\cdot}05$, Table III). Similar calculations for Group A rats alive at 85 weeks showed the incidence of hyperplasias to be not significant, if those associated with iatrogenic urolithiasis by week 27 were excluded (Table III).

Of the 3 bladder tumours detected in Group B, the simplest was found at 102 weeks: the bladder contained a small, spikey calculus, 2 mm in diameter, and the urothelium showed areas of focal, reactive, polyploid hyperplasia with Von Brunn's nests (Fig. 8*a*). Elsewhere, a few small papillomas were present, each consisting of a central capillary covered by well-differentiated, hyperplastic urothelium in which the cells displayed slight loss of polarity and pleomorphism (Fig. 8*b*). There was no epithelial invasion of the fibrovascular papillary core and the mitotic activity was up to 2 mitoses per high-power field. This tumour was therefore diagnosed as a papillary carcinoma *in situ*, Stage P.1.S., and Grade 1. The second bladder tumour, also found at 102

TABLE III.—*The incidence of urothelial lesions in the effective number of rats from 85 weeks*[a]

| Group | Treatment | Sex | Effective number[b] | Hyperplasias: Bladder | | Hyperplasias: Kidney | | Tumours: Bladder | Tumours: Kidney | Tumours: Ureter | Total |
|---|---|---|---|---|---|---|---|---|---|---|---|
| C | Nil | M | 47 | 2 | 2 | 1 | 3 | 0 | 0 | 0 | 0/82 |
| | | F | 35 (82) | 0 | | 2 | | 0 | 0 | 0 | |
| A | 2 g Saccharin/kg/ day (in water) | M | 64 | 6 | 10** | 10 | 19**** | 0 | 0 | 1 | 5/191* |
| | | F | 37 (101) | 4 | | 9 | | 0 | 1 | 0 | |
| B | 4 g Saccharin/kg/ day (in diet) | M | 49 | 6 | 11*** | 10 | 22***** | 3 | 0 | 0 | |
| | | F | 41 (90) | 5 | | 12 | | 0 | 0 | 0 | |

[a] When the first hyperplasias in the absence of crystalluria and also the first urothelial tumour (renal pelvis) were seen.

[b] Number of rats alive at 85 weeks (from Table I) and therefore at risk of developing urothelial hyperplasias or tumours.

* $P<0{\cdot}2$ Fischer exact probability test.

** $P<0{\cdot}1$
*** $P<0{\cdot}05$
**** $P<0{\cdot}01$
***** $P<0{\cdot}001$
} d.f. = 1

(a)

(b)

FIG. 8.—(a) Part of the bladder of a male Group B rat at 102 weeks in which a 2 mm calculus formed. The epithelium is thrown into hyperplastic, polypoid outgrowths penetrated by blood capillaries; downgrowths, or Vonn Brunn's nests, of disorientated cells are also formed. Haematoxylin and eosin. × 160. (b) Another area from the same bladder. Here, simple papillomas, each composed of a slender, fibrovascular core covered by well-differentiated urothelium, project into the vesical lumen. Haematoxylin and eosin. × 135.

weeks, showed papillary outgrowths from which circumscribed, nodular epithelial downgrowths invaded the submucosa. Irregularities in the basement membrane were occasionally present and suggested the escape of a few tumour cells (Fig. 9). In some areas, normally differentiated superficial cells at the urinary face could still be discerned and in others, sandy, mineralized deposits in the bladder lumen

FIG. 9.—Transitional-cell tumour in the bladder of a male Group B rat at 102 weeks. The epithelium forms a raised, nodular mass of polypoid outgrowths and solid, circumscribed downgrowths extending into the submucosa. Mitotic figures are present (arrows) and, at points (arrowheads) tumour cells appear to breach the basement membrane. Haematoxylin and eosin. × 180. (Reproduced by courtesy of *Int. Rev. Exp. Pathol.*, 1978, Academic Press.)

were in close contact with the tumour surface. The tumour was staged at P.1.a., with a papillary-plus-infiltrating growth pattern, a mitotic activity of up to 2 mitoses per high-power field and slight cellular pleomorphism (*i.e.* Grade 1). The third, and most advanced, bladder tumour (found at 95 weeks) was multifocal and included a pedunculated, papillary urothelial outgrowth and adjacent nodular areas of epithelial downgrowths into the lamina propria (Fig. 10*a*). The stromal core of the exophytic growth was invaded by epithelial nests (Fig. 10*b*) and the neoplastic areas were staged at P.1.a. Throughout, the tumour epithelium was composed of closely-packed, disorientated cells with darkly-staining, pleomorphic nuclei. Mitotic activity was prominent, with up to 3 mitoses per high-power field, Grade 2. In a few areas, sandy mineralization was present in the bladder lumen, close to the surface of the tumour.

(b) *Kidney*.—In the renal medulla, pyramid, papilla and fornix, calculi and/or mineralization were microscopically visible in all 3 groups, and were more frequent in untreated females than in untreated males (Table II). During the second year of the experiment, numerous microcalculi (small, crystalline deposits within or around the collecting tubules of the renal papilla) developed in saccharin-treated male and female rats of all groups, but more frequently in Group A, irrespective of ammonium chloride treatment. It is noteworthy that saccharin treatment caused a significant increase in renal microcalculi in males (Group A, $P<0{\cdot}001$; Group B,

(a)

(b)

FIG. 10.—(a) Part of a multifocal transitional-cell tumour in the bladder of a male Group B rat at 95 weeks. A pedunculated urothelial papilloma projects into the bladder lumen. Adjacent nodular urothelial downgrowths (arrows) extend into the oedematous mesenchyme. Sandy, mineralised deposits on the tumour surface have caused section scoring. Haematoxylin and eosin. ×45. (b) Enlargement of the papillary stem shown above. Nests of pleomorphic, disorientated transitional cells have invaded the fibrovascular core and adjacent bladder wall. Haematoxylin and eosin. ×125.

11

12

13

14

Fig. 11.—Renal fornix of a control male rat at 102 weeks. Mineralised foci have formed beneath and within the mildly hyperplastic urothelium that is focally eroded at the luminal surface (arrow). Some concretions and associated debris lie free in the renal pelvic lumen. Haematoxylin and eosin. ×110.

Fig. 12.—Longitudinal section through the kidney of a female Group A rat at 89 weeks. Branches of the *vasa recta* are dilated and congested with thrombi, some of which are beginning to calcify (arrows). Haematoxylin and eosin. ×16.

Fig. 13.—The renal fornix of a male Group A rat at 100 weeks, showing hyperplasia of the renal papilla and associated vascular telangiectasis. Haematoxylin and eosin. ×65.

Fig. 14.—Transitional cell tumour of the renal pelvis in a female Group A rat at 85 weeks. The neoplastic urothelium, composed of disorientated, pleomorphic cells and areas of squamous change, is penetrated by blood capillaries, and an early papillary process is featured. Haematoxylin and eosin. ×150.

$P<0\cdot01$) but not in females. Furthermore, the percentage incidence of microcalculi was higher in all Group A animals than in Group B, although the former received less saccharin, but in drinking water. In animals of all groups, sub- or intra-epithelial mineralized deposits were occasionally found in the renal pelvis, particularly at the fornix. In places, calculi were extruded into the pelvic lumen together with necrotic epithelium and erythrocytes (Fig. 11). Hyperplasia of the urothelium lining the renal pelvis was seen in all groups, but particularly in saccharin-treated ones, and usually appeared concurrently with hyperplasia of the bladder, *i.e.* after 85 weeks (Table III, $P<0\cdot01$, Group A; $P<0\cdot001$, Group B). In untreated rats minimal, 4-5-cell thick hyperplasias mainly involved the renal fornix, occasionally associated with sub-epithelial mineralized foci (Fig. 11). In saccharin-treated animals the hyperplasias were not only greater than in controls, but also frequently involved the epithelium of the renal papilla, often in conjunction with telangiectasia of branches of the *vasa recta* (Fig. 13, Table II, $P<0\cdot001$, Group A; $P<0\cdot05$, Group B). In this condition the blood vessels were dilated and congested with thrombi, some of which became calcified (Fig. 12). Such vascular changes were uncommon in control animals. In contrast, the number of animals showing hyalinization of kidney tubules and/or glomeruli was marked in controls and reduced in both saccharin-treated groups, notably in that receiving the higher dose of saccharin ($P<0\cdot001$).

A few kidney tumours of diverse histological appearance were found in treated and control rats of both sexes (Table II), including one transitional-cell tumour of the renal pelvis in a saccharin-treated female at 85 weeks (Group A). In this animal, the renal pelvic urothelium was grossly hyperplastic, of 15–20 cell layers, and in many places the underlying capillaries had proliferated into the epithelium (Fig. 14). Some areas were papillomatous or showed early squamous change, whilst others were characterized by marked cellular disorientation, pleomorphism and a mitotic activity of up to 2 mitoses per high-power field, Grade 2. The tumour was diagnosed as a papillary transitional-cell carcinoma. Although transitional-cell tumours, either of the bladder, ureter or kidney, were observed only in saccharin-treated animals, the combined incidence of all these tumours in the total "effective number" of rats given saccharin was not statistically significant (Table III, $P<0\cdot2$).

(c) *Other organs.*—Whilst the pathological changes recorded for other major organs were similar in all groups, there was a tendency for some organs, particularly the liver and spleen, in rats receiving saccharin to show a preponderance of lesions (Table II).

### DISCUSSION

To date, the evidence for the carcinogenicity of saccharin in experimental animals has appeared equivocal despite the variety of methods of administration and species and strains which have been used (see Introduction and Schmahl, 1973; Althoff *et al.*, 1975; Coulston *et al.*, 1975; Munro *et al.*, 1975; Kroes *et al.*, 1977). We have studied the effects on rats of high doses of saccharin given either in drinking water or in the diet both after life-time dosing and by examination of representative animals killed sequentially throughout the experiment. In particular, such studies should reveal progressive urothelial changes, such as hyperplasia and/or dysplasia, which might be expected to precede tumorigenesis, if saccharin behaves like most other urothelial carcinogens (Hicks & Chowaniec, 1978).

It is difficult to assess precisely the effect of saccharin on survival of the rats, since their mortality curves were somewhat altered by the programme of scheduled kills plus the unscheduled kills of sick animals in the second year of the experiment. However, the survival of all controls (Group C), particularly males, was as good as that reported elsewhere for untreated Wistar rats (*e.g.* Gaunt *et al.*,

1976). Despite the physiological disturbances and early deaths in males receiving saccharin in the drinking water, there was no significant difference in their mortality from the controls, possibly because their marginally reduced diet intake and consequent reduction in growth may have increased their life expectancy (Silberberg & Silberberg, 1955; Berg, 1967; Ross & Bras, 1971). There was a tendency for male rats receiving dietary saccharin to die before controls and, although not statistically significant, the results are in keeping with those of Munro *et al.* (1975), who observed an increased mortality in males with chronic dietary levels of saccharin above 2 g/kg/day. This may reflect a toxic or metabolic effect of high saccharin levels to which males are particularly susceptible.

The growth curves and urinary pH values together demonstrate that more physiological problems arise from administering saccharin in solution than if it is given, even at double the dose, in the diet. Thus, although some growth reduction was seen in all saccharin-treated animals, it was most marked in males receiving the sweetener in the drinking water. In these animals, not only was the liquid intake reduced, but there was also voluntary diet restriction; the two phenomena are known to be interdependent (Cizek & Nocenti, 1965). Rats receiving dietary saccharin also had a depressed growth rate even though there was no reduction in either food or water consumption. This confirms observations from other laboratories where saccharin was fed at levels above 2 g/kg/day (Munro *et al.*, 1975; Arnold *et al.*, 1978). The reduced calorific value of the diet and the treatment-related increased water content of the faeces may both have contributed to the lowered weight gain. Their concomitant increased fluid consumption, similarly found in other rats fed high dietary saccharin levels (Munro *et al.*, 1975; Arnold *et al.*, 1979) may have compensated for the mild diarrhoea, which suggests accumulation of osmotically active caecal material (Leegwater *et al.*, 1974).

The increased urinary alkalinity initially seen in many males in Group A could theoretically have resulted from inhibition of carbonic anhydrase activity in the kidney by the saccharin contaminant, OTS, since other sulphonamides show similar inhibitory properties (Krebs, 1948; Miller *et al.*, 1950; Levi *et al.*, 1971). Such a rise in the pH predisposes to urolithiasis by precipitating inorganic ions in the urine (Vermeulen *et al.*, 1951) and the resulting tendency to crystalluria in these males may have been exacerbated by their decreased fluid intake. However, no rise in urinary pH was seen in any Group B animals despite their higher OTS intake. Their increased fluid intake, which can lead to the production of copious, hypotonic urine (see Arnold *et al.*, 1979) apparently prevented alkalinization of the urine. Even so, male animals in this group showed an increased incidence of mineralization of the bladder and kidneys. Mineralization of the urinary tract, particularly in males, is also evident in other saccharin studies (Lessel, 1971; Taylor & Friedman, 1974), but it has been observed that males have a greater tendency than female rats to form urinary concretions in other experimental situations (Dunning *et al.*, 1947; Weil *et al.*, 1965). Moreover, the anatomy of the male bladder neck may favour stone retention (Casey *et al.*, 1978). A representative calculus from one of our saccharin-treated male rats was composed of magnesium ammonium phosphate, that is, struvite (infra-red analysis, courtesy of Dr J. W. Shaw, Boots Co. Ltd, England). Elevated urinary phosphate, magnesium and calcium levels have been reported in male rats fed high dietary levels of saccharin. These factors may well predispose to struvite formation but its precipitation may depend on urinary pH and volume (Toxicology Forum, 1978).

It is debatable whether the increase in urothelial hyperplasias found in saccharin-treated animals was directly attributable to the sweetener, or was a secondary response to the altered physiological state of the animal. It has been argued that any proliferative or carcinogenic effect of saccharin on the bladder may be attributed to iatrogenic urolithiasis (Lessel, 1971; National Research Council, 1974; Toxicology Forum, 1977). In male rats receiving saccharin in the drinking water, crystalluria may well have accounted for the hyperplasia we found before the addition of ammonium chloride, but urolithiasis was clearly not related to the late-developing hyperplasias in saccharin-treated rats, irrespective of how the sweetener was administered. Nevertheless, the 3 tumours in the bladders of saccharin-fed male rats were all associated with mineralization. In other studies also, bladder tumours occurred preferentially in male rats fed high levels of saccharin (Taylor & Friedman, 1974; Tisdel *et al.*, 1974; Arnold *et al.*, 1979) but there was no consistent relationship between urolithiasis and bladder tumours. Discrepancies in urolithiasis between different studies could reflect variation in dietary cation, carbohydrate or protein content, all of which may affect urine composition (Andersen, 1962; Lyon *et al.*, 1966; Woodard, 1971; Massry & Coburn, 1973). The urolithiasis associated with our 3 bladder tumours consisted of free-lying calculi and/or mineralized deposits adherent to or within the tumours. Free-lying calculi formed in the urine are known to predispose to bladder tumour formation (Chapman *et al.*, 1973) but whether the mineralized deposits that we found, some of which lie beneath the basal lamina, also contribute to tumorigenesis is debatable. In the latter situation the concretions could be a product of tissue necrosis which is concomitant with tumour growth. Distinction between the two types of calcification might assist interpretation of the conflicting published observations relating calcification to bladder tumours. Without this information it is impossible at present to assess the relevance of urolithiasis to urothelial tumour induction.

There is no consistent relationship between urothelial hyperplasias in the kidneys of saccharin-treated animals and kidney calcification. Although both the incidences of microcalculi and of renal pelvic urothelial hyperplasia in saccharin-treated animals were indeed greater than in controls, significantly so in the males, the incidence of intra- or sub-epithelial mineralization in the renal pelvis, a more likely source of epithelial irritation, was similar in treated and control groups. Furthermore, a urothelial tumour occurred in the renal pelvis of one female in which there was no such mineralization.

Of the other kidney lesions reported, telangiectasia of the *vasa recta* was a frequent finding in our saccharin-treated animals; comparable vascular changes have been reported in other saccharin-fed rats (Fitzhugh *et al.*, 1951; Arnold *et al.*, 1979). Analogous calcifying lesions and associated renal papillary hyperplasia are seen in rats fed modified starches, which are not reported to induce urothelial tumours (de Groot *et al.*, 1974). Saccharin is cleared from the kidney by active tubular secretion (Goldstein *et al.*, 1978) but this mechanism appears to be saturable in animals fed above 5% saccharin in the diet, producing accumulation of saccharin in the plasma and tissues (Toxicology Forum, 1978). Renal telangiectasis may thus indicate a pharmacological effect of saccharin, since, in those studies where it has been found, high levels of the sweetener were used. Alternatively, telangiectasis could form part of a neoplastic response in the kidney, since a similar condition, concomitant with hyperplasia of the renal papilla, is induced in the rat by methylnitrosourea (unpublished observations) and by the suspected renal carcinogen phenacetin (Johansson & Angervall, 1976). There was significantly less proteinuria or hyalinization of the renal tubules in the saccharin-treated animals than in control groups. Tubular hyalinization is commonly observed in ageing rats (Perry, 1965; Berg, 1967). The decreased incidence of this condition in saccharin-treated rats of both sexes may be related to their reduced body weights relative to controls, since reduced obesity can decrease nephrosis and tubular protein deposition (Saxton & Kimball, 1941; Simms, 1967).

The first suggestion that saccharin might be associated with neoplastic disease was made by Fitzhugh *et al.* (1951), who observed an increased incidence of lymphosarcomas in rats fed high levels of saccharin. Lymphoid tumours were more frequent in the saccharin-fed rats of Munro *et al.* (1975) and also in ours, particularly the males. In addition, extramedullary haematopoiesis in the spleen (SEH) was notable in females fed the higher dose of saccharin and, since this can develop before the appearance of both virally-induced lymphomas and spontaneous leukaemia in rodents (Boiron *et al.*, 1965; Siegler & Rich, 1964; Coleman *et al.*, 1977), SEH could indicate preneoplasia of the reticuloendothelial or haematopoietic tissues in our animals. However, the incidence of haematopoietic or lymphoid tumours in rats may be affected by other conditions, including reduced protein intake (Ross & Bras, 1973), increasing age, particularly in males (Swaen & Van Heerde, 1973) and oncogenic viruses (Pollard & Kajima, 1966). Whether high doses of saccharin affect the incidence of lymphoid tumours in rats by modifying the host response to such factors, or whether the sweetener has a more direct action on the reticuloendothelial/haematopoietic tissues, it is impossible to say at present.

Most evidence suggests that saccharin is hardly metabolized in the rat or monkey (Pitkin *et al.*, 1971; Kennedy *et al.*, 1972; Matthews *et al.*, 1973; Lethco & Wallace, 1975) and not at all in man (Byard *et al.*, 1974; Golberg, 1974). Although we found elevated incidences of hepatic zonal necrosis and proliferating periportal bile ducts in the saccharin-treated rats, neither saccharin nor the trace amounts of metabolites which are produced (o-sulphamoylbenzoic acid or ammonium *o*-carboxybenzenesulphonate) have been shown to be toxic in the dog or rat, even at high doses (Kennedy *et al.*, 1976). Nevertheless, saccharin is not entirely biologically inert; it is, for example, the most effective inhibitor, among a variety of structural analogues tested, of phosphotransferase and phosphohydrolase activities of glucose-6-phosphatase (Lygre, 1974). Furthermore, at concentrations estimated to be excreted in human urine, it significantly inhibits guanylate cyclase activity in various tissues, including the liver and urinary bladder (Vesely & Levey, 1978).

The evidence that saccharin is a solitary bladder carcinogen is still equivocal. Undoubtedly, an increase in urothelial hyperplasia is related to the saccharin treatment; in the mouse, similar slow-growing urothelial hyperplasia can be preneoplastic (Levi *et al.*, 1971). Saccharin clearly has some biological activity in rodents; more bladder tumours are found in rats fed saccharin than in those which are not, and, in both this study and others, the tumours occur predominantly in males after a long, often 2-year latent period (Lessel, 1971; Taylor & Friedman, 1974; Tisdel *et al.*, 1974; Arnold *et al.*, 1979). With the exception of 2 generation feeding studies (Taylor & Friedman, 1974; Tisdel *et al.*, 1974; Arnold *et al.*, 1979) however, in any single study the tumour incidence is too low to be statistically significant. Thus, the experimental evidence for saccharin as a solitary bladder carcinogen is inevitably controversial, for much larger numbers of animals than have been used so far are required to detect a low tumour incidence of statistical significance. Much epidemiological and experimental evidence (Peto, 1977) supports the hypothesis that carcinogenesis is a multifactorial or multistage process, involving initiation, promotion and propagation of tumour growth. Most powerful solitary carcinogens can initiate carcinogenesis, their metabolites can be demonstrated to interact with DNA and they are mutagenic in a variety of test systems. By contrast, saccharin does not appear to react with DNA (Lutz & Schlatter, 1977) and evidence for its mutagenicity in a variety of other test systems is debatable (*e.g.* Kramers, 1975;

Batzinger *et al.*, 1977; Stoltz *et al.*, 1977; Wolff & Rodin, 1978). If the low tumour incidence in saccharin-treated rodents, in this laboratory and in others, does reflect a direct biological effect of the saccharin, the sweetener is probably acting at a later stage in the neoplastic process than initiation.

There is already evidence that saccharin will promote tumour growth in an experimental system in which neoplasia has been deliberately initiated by a low dose of a known carcinogen (Hicks *et al.*, 1978) and this has recently been confirmed, both *in vivo* (Cohen *et al.*, 1978) and *in vitro* (Mondal *et al.*, 1978). We have discussed the possible relevance of saccharin's promoting activity to the human situation (Hicks & Chowaniec, 1977). Promoters characteristically produce hyperplasia and raise the mitotic index; both effects were seen in the urothelium of our saccharin-treated rats. Despite standard precautionary measures, laboratory animals may be exposed to trace amounts of chemical carcinogens, such as nitrosamine contaminants in the diet (*e.g.* from fishmeal), endogenously produced carcinogenic metabolites (*e.g.* tryptophan derivatives) or may even carry oncogenic viruses, any of which might produce the first initiating event in a multistage sequence of carcinogenesis. Most of the tumorigenic effects of saccharin could be accounted for on the assumption that it is promoting latent, or dormant, tumour cells already present in the experimental population. Thus, the equivocal results obtained with saccharin-treated rodents may reflect the problems inherent in attempting to assess the effect of a single variable in a multifactorial process.

The results presented in this paper demonstrate that when saccharin was administered in the drinking water, considerable physiological disturbances including iatrogenic urolithiasis ensued. Despite this, no bladder tumours occurred in these animals. As in other studies, bladder tumours developed only in animals treated with high doses of saccharin (*i.e.* at or above 5% of the diet). We have followed the histogenesis of urothelial changes by sequential sampling, and have shown that the low bladder-tumour incidence is superimposed on a background of urothelial hyperplasias which are significantly linked to the saccharin treatment. If the animals had been kept into their 3rd year, which would have been more analogous to the 7th and 8th decades in man, it is possible that sufficient time would have elapsed to allow any neoplastic potential of these slow-growing hyperplasias to be expressed.

This work was supported by a generous grant from the British Cancer Research Campaign. We are indebted to Drs R. C. B. Pugh and K. M. Cameron, Institute of Urology, St Paul's Hospital, London for consultation on the diagnoses of the urothelial tumours; to past and present members of the Department of Histopathology, Middlesex Hospital Medical School, London for qualified opinion on several incidental lesions; to Miss Leanora Simon for expert histological preparations, and to Misses Barbara Peach and Gwyneth Williams for invaluable secretarial assistance.

### REFERENCES

ALLEN, M. J., BOYLAND, E., DUKES, C. E., HORNING, E. S. & WATSON, J. G. (1957) Cancer of the urinary bladder induced in mice with metabolites of aromatic amines and tryptophan. *Br. J. Cancer*, **11**, 212.

ALTHOFF, J., CARDESA, A., POUR, P. & SHUBIK, P. (1975) A chronic study of artificial sweeteners in Syrian golden hamsters. *Cancer Lett.*, **1**, 21.

ANDERSEN, D. A. (1962) The nutritional significance of primary bladder stones. *Br. J. Urol.*, **34**, 160.

ARNOLD, D. L., MOODIE, C. A., GRICE, H. C. & 6 others (1979) Long term toxicity of orthotoluenesulphonamide and sodium saccharin in the rat. *Toxicol. Appl. Pharmacol.* (in press).

BATZINGER, R. P., OU, S-Y. L. & BUEDING, E. (1977) Saccharin and other sweeteners: mutagenic properties. *Science*, **198**, 944.

BERG, B. (1967) Longevity studies in rats: II. Pathology of ageing rats. In *Pathology of Laboratory Rats and Mice*. Eds E. Cotchin & F. J. C. Roe. Oxford: Blackwell. p. 749.

BOIRON, M., LEVY, J. P., LASNARET, J., OPPENHEIM, S. & BERNARD, J. (1965) Pathogenesis of Rauscher leukaemia. *J. Natl Cancer Inst.*, **35**, 865.

BRYAN, G. T., ERTURK, E. & YOSHIDA, O. (1970) Production of urinary bladder carcinomas in mice by sodium saccharin. *Science*, **168**, 1238.

BYARD, J. L., MCCHESNEY, E. W., GOLBERG, L. & COULSTON, F. (1974) Excretion and metabolism of saccharin in man. II. Studies with 14-C-labelled saccharin. *Fd Cosmet. Toxicol.*, **12**, 175.

CASEY, H. W., AYERS, K. M. & ROBINSON, F. R. (1978) The urinary system. In *Pathology of Laboratory Animals*. Eds K. Benirschke, F. M. Garner & T. C. Jones. New York: Springer-Verlag. p. 115.

CHAPMAN, W. H., KIRCHHEIM, D. & MCROBERTS, J. W. (1973) Effect of urine and calculus formation on the incidence of bladder tumours in rats implanted with paraffin wax pellets. *Cancer Res.*, **33**, 1225.

CIZEK, L. J. & NOCENTI, M. R. (1965) Relationship between water and food ingestion in the rat. *Am. J. Physiol.*, **208**, 615.

CLARKE, H. E., COATES, M. E., EVA, J. K. & 5 others (1977) Dietary standards for laboratory animals. *Lab. Animals*, **11**, 1.

COHEN, S. M., ARAI, M. & FRIEDELL, G. H. (1978) Promoting effect of DL-tryptophan and saccharin in urinary bladder carcinogenesis in the rat. *Proc. Am. Assoc. Cancer Res.*, **19**, 4.

COLEMAN, G. L., BARTHOLD, S. W., OSBALDISTON, G. W., FOSTER, S. J. & JONAS, A. M. (1977) Pathological changes during ageing in barrier-reared Fischer 344 male rats. *J. Gerontol.*, **32**, 258.

COULSTON, F., MCCHESNEY, E. W. & GOLBERG, L. (1975) Long-term administration of artificial sweeteners to the Rhesus monkey (*M. mulatta*). *Fd Cosmet. Toxicol.*, **13**, 297.

DE GROOT, A. P., TIL, H. P., FERON, V. J., DREEF-VAN DER MEULEN, H. C. & WILLEMS, M. I. (1974) Two-year feeding and multigeneration studies in rats on five chemically modified starches. *Fd Cosmet. Toxicol.*, **12**, 651.

DUNNING, W. F., CURTIS, M. R. & SEGALOFF, A. (1947) Strain differences in response to diethylstilboestrol and the induction of mammary gland and bladder cancer in the rat. *Cancer Res.*, **7**, 511.

EGEBERG, R. O., STEINFELD, J. L., FRANTZ, I. & 5 others (1970) Report to the secretary of HEW from the Medical Advisory Group on Cyclamates. *J. Am. Med. Assoc.*, **211**, 1358.

FITZHUGH, O. G., NELSON, A. A. & FRAWLEY, J. P. (1951) Comparison of chronic toxicities of synthetic sweetening agents. *J. Am. Pharm. Assoc.*, **40**, 583.
Further Data from these experiments are presented In *Cancer Testing Technology and Saccharin*, p. 60. Office of Technology Assessment, US Govt. Printing Office, Washington, D.C.

FLAKS, A. & CLAYSON, D. B. (1975) The influence of ammonium chloride on the induction of bladder tumours by 4-ethylsulphonylnaphthalene-1-sulphonamide. *Br. J. Cancer*, **31**, 585.

GAUNT, I. F., HARDY, J., GRASSO, P., GANGOLLI, S. D. & BUTTERWORTH, K. R. (1976) Long-term toxicity of cyclohexylamine hydrochloride in the rat. *Fd Cosmet. Toxicol.*, **14**, 255.

GOLBERG, L. (1974) Artificial sweeteners. In *Safety of Saccharin and Sodium Saccharin in the Human Diet.* Washington, D.C.: Nat. Acad. Sci., PB **238-137**, p. 25.

GOLDSTEIN, R. S., HOOK, J. B. & BOND, J. T. (1978) Renal tubular transport of saccharin. *J. Pharmacol. Exp. Therapeutics.*, **204**, 690.

HICKS, R. M. & CHOWANIEC, J. (1977) The importance of synergy in the induction of bladder cancer in experimental animals and humans. *Cancer Res.*, **37**, 2943.

HICKS, R. M. & CHOWANIEC, J. (1978) Experimental induction, histology and ultrastructure of hyperplasia and neoplasia of the urinary bladder. *Int. Rev. Exp. Pathol.*, **18**, 199.

HICKS, R. M., CHOWANIEC, J. & WAKEFIELD, J. St. J. (1978) The experimental induction of bladder tumours by a two-stage system. In *Mechanisms of Tumour Promotion and Cocarcinogenesis.* Vol 2. Ed T. Slaga. New York: Raven Press. p. 475

HICKS, R. M., WAKEFIELD, J. St. J. & CHOWANIEC, J. (1973) Impurities in saccharin and bladder cancer. *Nature*, **243**, 424.

JOHANSSON, S. & ANGERVALL, L. (1976) Urothelial changes of the renal papilla in Sprague-Dawley rats induced by feeding phenacetin. *Acta. Pathol. Microbiol. Scand.* A., **84**, 375.

KENNEDY, G. L., FANCHER, O. E. & CALANDRA, J. C. (1976) Subacute toxicity studies with sodium saccharin and two hydrolytic derivatives. *Toxicology*, **6**, 133.

KENNEDY, G. L., FANCHER, O. E., CALANDRA, J. C. & KELLER, R. E. (1972) Metabolic fate of saccharin in the albino rat. *Fd Cosmet. Toxicol.*, **10**, 143.

KRAMERS, P. G. M. (1975) The mutagenicity of saccharin. *Mutation Res.*, **32**, 81.

KROES, R., PETERS, P. W. J., BERKVENS, J. M., VERSCHUUREN, H. G., DE VRIES, TH. & VAN ESCH, G. J. (1977) Long-term toxicity and reproduction study (including a teratogenicity study) with cyclamate, saccharin and cyclohexylamine. *Toxicology*, **8**, 285.

KREBS, H. A. (1948) Inhibition of carbonic anhydrase by sulphonamides. *Biochem. J.*, **43**, 525.

LEEGWATER, D. C., DE GROOT, A. P. & VAN KALMTHOUT-KUYPER, M. (1974) The aetiology of caecal enlargement in the rat. *Fd Cosmet. Toxicol.*, **12**, 687.

LESSEL, B. (1971) Carcinogenic and teratogenic aspects of saccharin. *Proc. III Int. Cong. Fd Sci. Technol.* 1970. Eds G. F. Stewart & C. L. Willey. Chicago: Inst. Food Technologists. p. 764.

LETHCO, E. J. & WALLACE, W. C. (1975) The metabolism of saccharin in animals. *Toxicology*, **3**, 287.

LEVI, P. E., KNOWLES, J. C., COWEN, D. M., WOOD, M. & COOPER, E. H. (1971) Disorganisation of mouse bladder epithelium induced by 2-acetylaminofluorene and 4-ethylsulphonylnaphthalene-1-sulphonamide. *J. Natl Cancer Inst.*, **46**, 337.

LUTZ, W. K. & SCHLATTER, C. H. (1977) Saccharin does not bind to DNA of liver or bladder in the rat. *Chem.-Biol. Interact.*, **19**, 253.

LYGRE, D. G. (1974) The inhibition by saccharin and cyclamate of phosphotransferase and phosphohydrolase activities of glucose-6-phosphatase. *Biochim. Biophys. Acta*, **341**, 291.

LYON, E. S., BORDEN, T. A., ELLIS, J. E. & VERMEULEN, C. W. (1966) Calcium oxalate lithiasis produced by pyridoxine deficiency and inhibition with high magnesium diets. *Invest. Urol.*, **4**, 133.

MASSRY, S. G. & COBURN, J. W. (1973) The hormonal and non-hormonal control of renal excretion of calcium and magnesium. *Nephron.*, **10**, 66.

MATTHEWS, H. B., FIELDS, M. & FISHBEIN, L. (1973) Saccharin: distribution and excretion of a limited dose in the rat. *J. Agric. Food Chem.*, **21**, 916.

MILLER, W. H., DESSERT, A. M., & ROBLIN, R. O. (1950) Heterocyclic sulphonamides as carbonic anhydrase inhibitors. *J. Am. Chem. Soc.*, **72**, 4893.

MONDAL, S., BRANKOW, D. W. & HEIDELBERGER, C. (1978) Enhancement of oncogenesis in C34/10T1/2 mouse embryo cell cultures by saccharin. *Science*, **201**, 1141.

MUNRO, I. C., MOODIE, C. A., KREWSKI, D. & GRICE, H. C. (1975) A carcinogenicity study of commercial saccharin in the rat. *Toxicol. Appl. Pharmacol.*, **32**, 513.

NATIONAL RESEARCH COUNCIL (1974) Report to the FDA on the *Safety of Saccharin and Sodium Saccharin in the Human Diet.* Publ. no. **238-137.** Springfield, Va.: National Technical Information Service.

PERRY, S. W. (1965) Proteinuria in the Wistar rat. *J. Pathol. Bacteriol.*, **89**, 729.

PETO, R. (1977) Epidemiology, multistage models and short-term mutagenicity tests. In *Origins of Human Cancer.* Book C. Human Risk Assessment. Eds H. H. Hiatt, J. D. Watson & J. A. Winsten. Cold Spring Harbor Conf. Cell Proliferation. Vol 4. p. 1403.

PETO, R., PIKE, M. C., ARMITAGE, P. & 7 others (1977) Design and analysis of randomised clinical trials requiring prolonged observation of each patient. II. Analysis and examples. *Br. J. Cancer*, **35**, 1.

PITKIN, R. M., ANDERSEN, D. W., REYNOLDS, W. A. & FILER, L. J. (1971) Saccharin metabolism in *Macaca mulatta. Proc. Soc. Exp. Biol. Med.*, **137**, 803.

POLLARD, M. & KAJIMA, M. (1966) Leukaemia in germ-free rats. *Proc. Soc. Exp. Biol. Med.*, **121**, 585.

PRICE, J. M., BIAVA, C. G., OSER, B. L., VOGIN, E. E., STEINFELD, J. J. & LEY, H. L. (1970) Bladder tumours in rats fed cyclohexylamine or high doses of a mixture of cyclamate and saccharin. *Science*, **167**, 1131.

PUGH, R. C. B. (1973) The pathology of cancer of the bladder. An editorial overview. *Cancer*, **32**, 1267.

ROE, F. J. C., LEVY, L. S. & CARTER, R. L. (1970) Feeding studies on cyclamate, saccharin and sucrose for carcinogenic and tumour-promoting activity. *Fd Cosmet. Toxicol.*, **8**, 135.

ROSS, M. H. & BRAS, G. (1971) Lasting influence of early caloric restriction on the prevalence of neoplasms in the rat. *J. Natl Cancer Inst.*, **47**, 1095.

ROSS, M. H. & BRAS, G. (1973) Influence of protein under- and overnutrition on spontaneous tumour prevalence in the rat. *J. Nutr.*, **103**, 944.

SAXTON, J. A. & KIMBALL, G. C. (1941) Relation of nephrosis and other diseases of albino rats to age and to modification of the diet. *Archs. Pathol.*, **32**, 951.

SCHMAHL, VON D. (1973) Fehlen einer kanzerogenen Wirkung von Cyclamat Cyclohexylamin und Saccharin bei Ratten. *Arzneim.-Forsch.*, **23**, 1466.

SIEGEL, S. (1956) The Fisher Exact Probability Test. In *Nonparametric Statistics for the Behavioural Sciences.* Tokyo: Kogukusha Co. p. 96.

SIEGLER, R. & RICH, M. A. (1964) Comparative pathogenesis of murine viral lymphoma. *Cancer Res.*, **24**, 1406.

SILBERBERG, M. & SILBERBERG, R. (1955) Diet and lifespan. *Physiol. Revs.*, **35**, 347.

SIMMS, H. S. (1967) Longevity studies in rats. I. Relation between lifespan and age of onset of specific lesions. In *Pathology of Laboratory Rats and Mice.* Eds E. Cotchin & F. J. C. Roe. Oxford: Blackwell. p. 733.

STOLTZ, D. R., STAVRIC, B., KLASSEN, R., BENDALL, R. D. & CRAIG, J. (1977) The mutagenicity of saccharin impurities. I. The detection of mutagenic activity. *J. Environ. Pathol. Toxicol.*, **1**, 139.

SWAEN, G. J. V. & VAN HEERDE, P. (1973) Tumours of the haematopoetic system. In *WHO/IARC Pathology of Tumours in Laboratory Animals. Vol 1. Tumours of the Rat.* Part 1. Ed V. S. Turusov. Lyon: IARC Scient. Publ, No. 5. p. 185.

TAYLOR, J. D., RICHARDS, R. K. & WEIGAND, R. G. (1968) Toxicological studies with sodium cyclamate and saccharin. *Fd Cosmet. Toxicol.*, **6**, 313.

TAYLOR, J. M. & FRIEDMAN, L. (1974) Combined chronic feeding and three-generation reproduction study of sodium saccharin in the rat. *Toxicol. Appl. Pharmacol.*, **29**, 154 (Abst.).
Further data from these experiments are presented in: *Cancer Testing Technology and Saccharin*, p. 53-57. Washington, D.C. 1977. Office of Technology Assessment, US Govt. Printing Office.

TILTMAN, A. J. & FRIEDELL, G. (1971) The histogenesis of experimental bladder cancer. *Invest. Urol.*, **9**, 218.

TISDEL, M. O., NEES, P. O., HARRIS, D. L. & DERSE, P. H. (1974) Long-term feeding of saccharin in rats. In *Symposium Sweeteners.* Ed G. E. Inglett. Westport: Avi· p. 145.
Further data from these experiments are presented in: *Cancer Testing Technology and Saccharin*, p. 57-60. Washington, D. C. 1977. Office of Technology Assessment, US Govt. Printing Office.

TOXICOLOGY FORUM (1977) Saccharin: Current Scientific Status and Future Research Needs. Key Biscayne, Florida.

TOXICOLOGY FORUM (1978) *Saccharin Update.* Special Session Proceedings, Aspen, Colorado, p. 50.

VERMEULEN, C. W., RAGINS, H. D., GROVE, W. J. & GOETZ, R. (1951) Experimental urolithiasis: III. Prevention and dissolution of calculi by alteration of urinary pH. *J. Urol.*, **66**, 1.

VESELY, D. L. & LEVEY, G. S. (1978) Saccharin inhibits guanylate cyclase activity: possible relationship to carcinogenesis. *Biochem. Biophys. Res. Commun.*, **81**, 1384.

WEIL, C. S., CARPENTER, C. P. & SMYTH, H. F. (1965) Urinary bladder response to diethylene glycol. *Arch. Environ. Health*, **11**, 569.

WHO/IARC *Pathology of Tumours in Laboratory Animals.* Vols I and II. Part 1, 1973; Part 2, 1976. Ed V. S. Turusov. Lyon: IARC Scientific Publications.

WOLFF, S. & RODIN, B. (1978) Saccharin-induced sister chromatid exchanges in Chinese hamster and human cells. *Science*, **200**, 543.

WOODARD, J. C. (1971) Relationship between the ingredients of semipurified diets and nutritional nephrocalcinosis. *Am. J. Pathol.*, **65**, 269.

YATES, F. (1934) Contingency tables involving small numbers and the Chi-square test. *J. R. Statis. Soc.*, Suppl. **1**, 217.